# Human mitochondrial DNA lineages in Iron-Age Fennoscandia suggest incipient admixture and eastern introduction of farming-related maternal ancestry

**DOI:** 10.1038/s41598-019-51045-8

**Published:** 2019-11-15

**Authors:** Sanni Översti, Kerttu Majander, Elina Salmela, Kati Salo, Laura Arppe, Stanislav Belskiy, Heli Etu-Sihvola, Ville Laakso, Esa Mikkola, Saskia Pfrengle, Mikko Putkonen, Jussi-Pekka Taavitsainen, Katja Vuoristo, Anna Wessman, Antti Sajantila, Markku Oinonen, Wolfgang Haak, Verena J. Schuenemann, Johannes Krause, Jukka U. Palo, Päivi Onkamo

**Affiliations:** 1 Department of Biosciences, University of Helsinki, Helsinki, Finland; 2 Department of Archaeogenetics, Max Planck Institute for the Science of Human History, Jena, Germany; 3 Institute for Archaeological Sciences, Archaeo- and Palaeogenetics, University of Tübingen, Tübingen, Germany; 4 Department of Cultures, University of Helsinki, Helsinki, Finland; 5 Laboratory of Chronology, Finnish Museum of Natural History, University of Helsinki, Helsinki, Finland; 6 Peter the Great Museum of World Anthropology and Ethnography (Kunstkamera), Russian Academy of Science, St. Petersburg, Russia; 7 Department of Archaeology, University of Turku, Turku, Finland; 8 Finnish Heritage Agency, Helsinki, Finland; 9 Department of Forensic Medicine, University of Helsinki, Helsinki, Finland; 10 Institute of Evolutionary Medicine, University of Zürich, Zürich, Switzerland; 11 Forensic Genetics Unit, National Institute for Health and Welfare, Helsinki, Finland; 12 Department of Biology, University of Turku, Turku, Finland

**Keywords:** Evolutionary genetics, Population genetics

## Abstract

Human ancient DNA studies have revealed high mobility in Europe’s past, and have helped to decode the human history on the Eurasian continent. Northeastern Europe, especially north of the Baltic Sea, however, remains less well understood largely due to the lack of preserved human remains. Finland, with a divergent population history from most of Europe, offers a unique perspective to hunter-gatherer way of life, but thus far genetic information on prehistoric human groups in Finland is nearly absent. Here we report 103 complete ancient mitochondrial genomes from human remains dated to AD 300–1800, and explore mtDNA diversity associated with hunter-gatherers and Neolithic farmers. The results indicate largely unadmixed mtDNA pools of differing ancestries from Iron-Age on, suggesting a rather late genetic shift from hunter-gatherers towards farmers in North-East Europe. Furthermore, the data suggest eastern introduction of farmer-related haplogroups into Finland, contradicting contemporary genetic patterns in Finns.

## Introduction

Genetic studies on anthropological remains have exceedingly helped to shed light on various human populations as well as past events and processes. These include for instance the initial colonization of Europe by modern humans c. 40 kya^1,2^, the Holocene hunter-gatherer communities^1,3^, the major population turnover associated with the Neolithic spread of agriculture from Anatolia^3–5^, and the massive Bronze-Age influx of genes, culture and customs of the Yamnaya-related people into Europe from the Pontic-Caspian steppe^6,7^.

In terms of mitochondrial DNA (mtDNA), these cultural turnovers and population migrations in Europe involved also changes in the haplogroup composition. In hunter-gatherer populations the dominating mitochondrial lineage has been U, especially its subgroups U4, U5a and U5b^3^. In the advent of the Neolithic revolution, these U subgroups were largely supplanted by farmer-associated haplogroups H, HV, J, K, N1a, T2 and W^4,5^. The subsequent spread of Yamnaya-related people and Corded Ware Culture in the late Neolithic and Bronze Age were accompanied with the increase of haplogroups I, U2 and T1 in Europe (See^8^ and references therein).

Whereas the ancient DNA (aDNA) composition and its changes in mainland Europe are increasingly well understood, northeastern Europe has been far less studied. The oldest human DNA analyzed in this region derive from the Mesolithic burial sites in Huseby Klev, western Sweden (9800 calBP^9^), Hummervikholmen in Norway (9300 calBP^10^) and Yuznuy Olennij Ostrov on Lake Onega, Russia (~8400 calBP^7,11^). In addition, Mesolithic to Bronze-Age DNA data from several sites in the Baltic countries have been published^6,12–14^.

Despite the relatively close geographical proximity, little is known about the ancient DNA diversity in regions immediately north of the Baltics. This is largely due to the scarcity of preserved anthropological remains. In the hemiboreal forest zone the soil pH, together with annual freeze-thaw cycles has highly detrimental effects on bone material to the extent to which no unburnt remains older than ~2000 years exist^15^. However, archaeological evidence strongly suggests that the most notable colonization events in the region have taken place much earlier^16,17^. Consequently, the lack of archaeological bone material gravely limits the capability of aDNA studies in resolving the human population history of the Taiga belt and the processes that have shaped present-day diversity. Despite these shortcomings, aDNA has recently been recovered from c. 1500 year-old bones from Levänluhta in western central Finland^18,19^. Genomic data from these samples show a Siberian ancestry component still prominently present today, particularly in the indigenous Saami people, and to a lesser extent in modern Finns. Although these data suggest a widespread presence of genetically Saami-like people around eastern Fennoscandia during the Iron Age, more wide-spread sampling in space and time is necessary for understanding the past population dynamics, and emerging of the contemporary genetic diversity in Finland.

In terms of both genetics and culture, modern Finns show a unique combination of eastern and western European elements, which most likely reflects the settlement history. The first archaeological evidence of human presence in Finland dates relatively late to c. 11000–9000 years ago, soon after the continental ice sheet retreated. According to the archaeological record, the region has since supported a continuous human occupation until today^17^. Several influential waves of material culture have been shown to extend into Finland: the first one brought the Sperrings or Säräisniemi pottery to the area c. 7500 years ago^20^, the second presented the typical Comb-Ceramic at 6000 years ago^16,17^, one of the most influential prehistoric cultures in the wider region. Finally, around 4700 years ago, the Corded-Ware culture reached Finland^16,17,21^. The most significant cultural changes, possibly driven by expansions, proceeded from east/south-east and extended into most of today’s territory of Finland, while the Corded-Ware culture influence spread from south occupying only the southwestern part of Finland. Later, Bronze Age brought an increase of human activity^17^ and coinciding advance of cereal cultivation^22^. Finland also saw a pronounced Scandinavian impact along the western coast, while the inland was dominated by eastern influences, seen e.g. in the arrival of ferrous metallurgy c. 500 BC and stylistic features of Bronze-Age and Iron-Age items^21^. Iron Age in Finland starts around 500 BC and continues until the end of Crusade period (*c*. 1200 AD in west and 1300 AD in east). Unlike most of the Europe, the Middle Ages starts as late as 1200 AD in western parts of country and 1300 AD in east and shifts into Early modern period in the beginning of 16^th^ century (for a review of archaeological and historical periods in Finland see^21^).

Linguistically, contemporary Finns and Saami differ from most other Europeans in speaking a Uralic language, unrelated to the majority of European languages, which belong to the Indo-European language family. Finns are also genetically distinct from their neighboring populations and form outliers in the genetic variation within Europe^23^. This genetic uniqueness derives from both reduced genetic diversity^24,25^ and an Asian influence to the gene pool^24^. Within Finland, an unusually strong genetic border bisects the population along a northwest to southeast axis^24,26,27^, and is interpreted to reflect an ancient boundary between hunter-gatherer and farmer populations^28^. The expanse of agriculture north-east of this border was probably limited by environmental factors, especially the length of the growing season. Later, this border has most likely acted in demarcating the spread of western and eastern political and cultural impacts influencing the placement of first political border between Sweden and Novgorod through the middle of Finland (Treaty of Noteborg 1323 AD).

**Figure 1:**
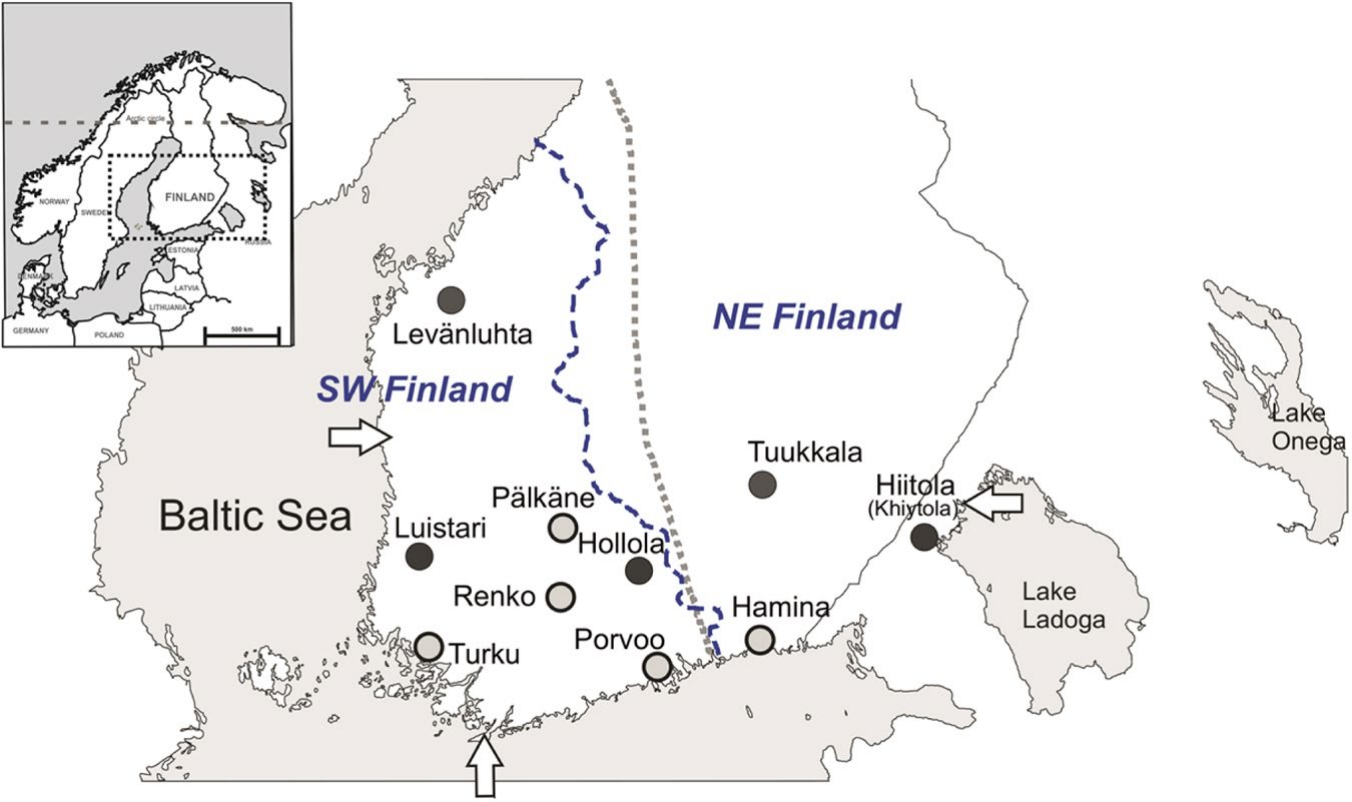
Map of Finland and the burial sites presented in this study. Iron-Age and medieval sites (Levänluhta, Luistari, Hollola, Hiitola and Tuukkala) are marked with dark grey circles. Early-modern and modern sites (Pälkäne, Porvoo, Renko, Turku and Hamina) are marked with light grey circles. Arrows indicate three reference points (Hanko, Uusikaupunki and Lahdenpohja) used in the multinomial logistic regression analysis (see Section 2.7). Small map of northern Europe is modified from Neuvonen *et al*.^28^. Blue dashed line represents the border of southwestern (SW) and northeastern (NE) subpopulations of contemporary Finns (Neuvonen *et al*.^28^ was used as a reference for this border). Grey dotted line represents the boundary of Finnish folk culture between western and northeastern Finland described in Talve 2000^74^.

In order to gain better insight into the genetic history of Finns, we here describe 103 complete mitochondrial genomes reconstructed from bone samples from ten burial sites in southern Finland and the Republic of Karelia, Russia (former Finnish territory; Fig. 1). The main focus is on the 70 complete mitochondrial genomes from five archaeological burial sites in Finland spanning spatially from western coast to Lake Ladoga, and Late Roman Iron Age (300 AD) to the Middle Ages (1500 AD) (Tables 1, S1 and Supplementary Material S1). In addition, we include 33 mitochondrial genomes from later, mainly historical burials (1400–1800 AD) from five sites across southern Finland. While mtDNA genomes of Iron-Age and Early-Medieval Finland cannot be used to directly target questions about the colonization of Finland, they provide a spatio-temporal transect to the maternal ancestry of the early inhabitants in this region, and help to understand patterns observed in Finland’s modern mtDNA diversity.

**Table 1:** Sites presented in this study.

Site	Archaeological dating (AD)*	Period based on archaeological dating	Number of individuals with^14^C date	^14^C (calAD)**	Number of individuals sampled	Number of individuals in mtDNA capture	Number of complete mtDNA sequences obtained	Number of complete mtDNA sequences used in statistical analyses
Levänluhta	300–800	Roman Iron-Age, Merovingian	4	370–720	13	13	12	12
Luistari	600–1300	Merovingian, Viking, Crusade	10	760–1295	25	21	10	10
Hollola	1050–1400	Crusade	11	955–1390	20	18	16	14
Hiitola	1200–1500	Crusade, medieval	10	1170–1470	16	16	14	13
Tuukkala	1200–1400	Crusade, medieval	4	1225–1525	30	30	18	17
Pälkäne	1200–1700	Crusade, medieval, post-medieval	4	1280–1640	4	4	4	4
Porvoo	1300–1900	medieval, post-medieval	5	1610–1825	9	9	7	7
Renko	1500–1800	medieval, post-medieval	3	NA	8	8	8	8
Turku	1550–1650	post-medieval	NA	NA	5	5	5	4
Hamina	1700–1800	post-medieval	NA	NA	9	9	9	9
TOTAL			47		141	134	103	98

^*^Dating based on the burial contexts.

**Defined based on individual’s ^14^C dates with Oxcal commands ‘Phase/Boundary’. Phase boundaries provide estimate for starts and ends of the site usages. Phase boundaries are here defined for sites with four or more individuals with ^14^C dates. The mean values are given. Radiocarbon dates include datings presented in this study and datings published elsewhere. See Section 2.2, Supplementary Material S2 and Supplementary Table S2 for more detailed information.

## Results

### Authenticity of ancient-DNA results

Based on the shotgun sequencing, out of the total of 141 individuals sampled, 134 were included in mitochondrial capture. Mitochondrial genomes for 103 individuals passed the quality control thresholds, while 31 samples were excluded from further analyses due to insufficient data (less than fivefold mitochondrial coverage) or high contamination levels (Supplementary Table S1). Ancient-DNA yield for all 103 samples was studied with several criteria of authentication. All samples showed fragment sizes ranging between 40–250 bp, as expected for ancient DNA^29^. Fragments under 30 bp were filtered out as a mapping quality control. All samples had an average fragment length of 47 to 95 bp. The authentic ancient DNA is often fragmented compared to the modern DNA, and fragments as short as 50–65 bp are common. The samples included in the downstream analyses yielded between 1426 and 395345 unique human mitochondrial fragments with an average coverage ranging from 5-fold to 1683-fold. The first-base damage on the fragments varied between 5–36% on the 3′-end and 4–34% on the 5′-end. Previous studies have proven that cytosine deamination is influenced by the age of the sample^30,31^ and the mean temperature of the site^31^. Considering the climatic conditions in Finland, e.g., low mean temperature, and the relatively young age especially for the post-medieval samples, 3′ and 5′ damage values below 5% are plausible. No samples were therefore omitted from the study based on these criteria.

The contamination rates of the 103 samples were further evaluated by Schmutzi^32^. 36 samples had Schmutzi contamination estimates exceeding 5% and were excluded (Supplementary Table S1). The remaining samples were then analyzed with ContamMix^33^: the resulting crude contamination estimates as well as the *a posteriori* estimates of contamination along with their 95% confidence intervals (CI) from the MCMC are reported in Supplementary Table S1. The CIs ranged from 0% to 17.2%; in ten cases they exceeded 10%, even though estimates by Schmutzi had remained below 5%. These cases were visually inspected with Geneious 11.0.3 (www.geneious.com). For each of them, the majority call supported the previously assigned haplogroup.

### Radiocarbon datings

For this study, we report new ^14^C dates for 42 individuals (Supplementary Table S1). Radiocarbon dates for nine individuals were determined previously (see Supplementary Table S1). Based on radiocarbon dates and/or dating of the context, the studied burial sites cover the timespan from the Roman Iron Age (300 AD) to historical times (19th century). For sites Levänluhta, Luistari, Hollola, Hiitola, Tuukkala, Pälkäne and Porvoo the highest posterior densities (HPD) for site’s start and end boundaries were determined. The mean values for obtained phase boundaries are presented in Table 1, and 68% and 95% HPD regions are presented in Supplementary Table S2 and in Supplementary Fig. S1. Intervals for mean values of boundaries obtained based on radiocarbon dates were in accordance with dates determined based on the archaeological context (Table 1).

### MtDNA data and haplotypic variation

A total of 95 unique complete-mitogenome haplotypes were observed among the 103 complete sequences retrieved: three haplotypes were shared between sampling sites and five within a site. In the latter cases, the placement of the skeletal samples suggests that the shared haplotypes have been carried by different individuals, who may have been maternally related: identical haplotypes (haplogroup U5a2a1e) were obtained from remains of a c. 5-year-old child (grave 18, TU666) and an older woman (grave 7, TU655) from Hollola. Identical haplotypes (haplogroup H85) were also observed in a middle-aged adult (grave 6, TU661) and a c. 18-month-old child (grave 15, TU668) from Hollola. At the Hiitola site, identical haplotypes (haplogroup W6) were shared between two individuals from distinct graves (individual TU566 from grave 80 and individual TU675 from grave 30). At the Tuukkala site, two individuals showed identical haplotypes (haplogroup H10e, individuals TU631 and TU645). At Turku, two adults shared the same haplotype belonging to the basal haplogroup H (samples TU582 and TU588). Haplotypes for 103 individuals are presented in Supplementary Table S3.

As the subsequent statistical methods assume that samples derive from unrelated individuals, five samples - one of each identical haplotype pairs within sites (TU666, TU668, TU675, TU645 and TU588) - were removed from the subsequent analyses due to their possible maternal relatedness.

The mean number of pairwise differences, calculated from complete mitochondrial genomes, was highest within Porvoo (MNPD = 33.7 ± 16.8) and lowest within Renko (MNPD = 21.8 ± 10.8) (Supplementary Table S4). Due to the small number of individuals per site and utilization of unique complete mtDNA sequences, haplotype diversities (H) were relatively high (with mean 1.0 and standard deviation ranking from 0.0202 to 0.1768).

### MtDNA haplogroup composition at the ancient sites

**Figure 2:**
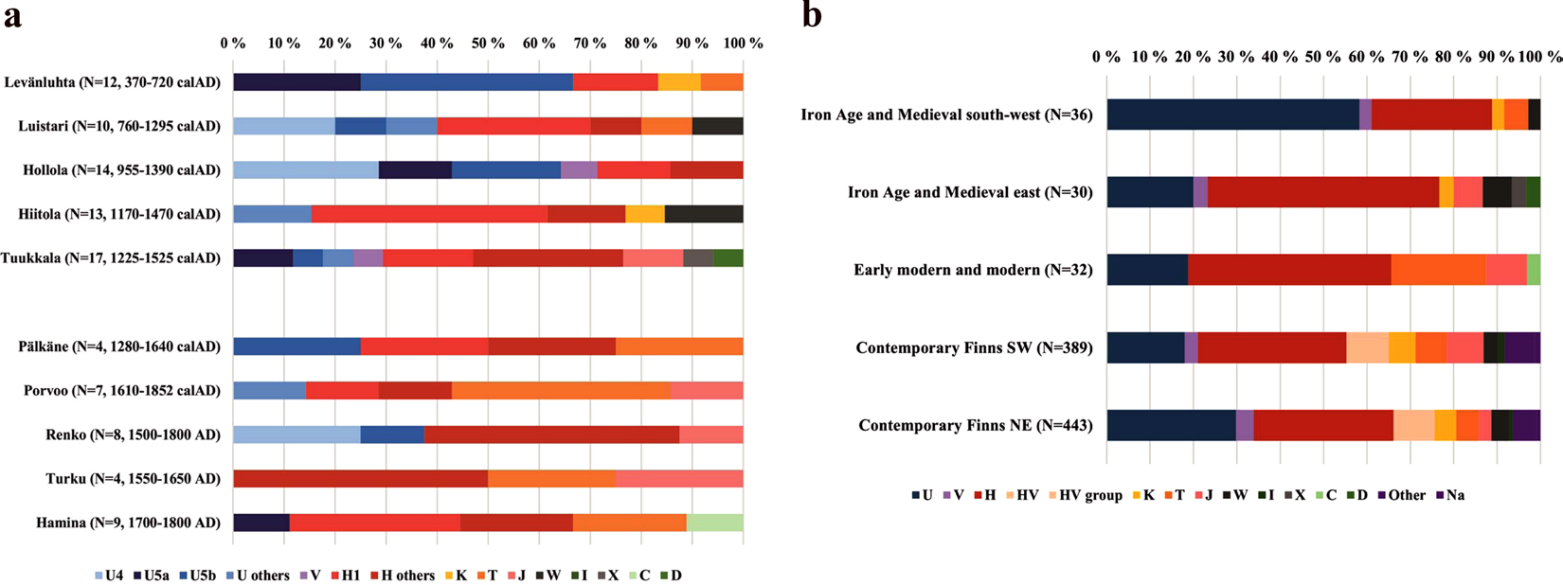
(**a**) MtDNA haplogroup distribution at each site. Only unique haplotypes per site are included. Ages of the sites are presented based on the interval for mean values of phase boundaries for start and end distribution when available (i.e. for Levänluhta, Luistari, Hollola, Hiitola, Tuukkala, Pälkäne, and Porvoo, see Section 2.2.) and based on archaeological context for other sites. (**b**) MtDNA haplogroup frequencies when pooled according to chronological and geographical criteria. Only unique haplotypes within site are included. Iron-Age and medieval south-west includes Levänluhta, Hollola and Luistari; Iron-Age and medieval east includes Hiitola and Tuukkala; Early modern and modern includes Pälkäne, Porvoo, Renko; Frequencies for contemporary Finns from^28^.

Burial site-specific haplogroup frequencies of the 98 complete mitochondrial sequences showed considerable between-site variation (Fig. 2 and Supplementary Table S5). The observed frequencies of the main haplogroups in the whole dataset resembled the prevalence among contemporary Finns. As today, haplogroups U and H were the most common, yet with slightly higher overall frequencies than today (U 33.7% vs. 24.1%, and H 41.8% vs. 33.2%). However, when grouped temporally into Iron-Age and medieval sites (IAM) and early-modern and modern sites (EMM), differences were observed: the IAM sites (i.e., Levänluhta, Luistari, Hollola, Hiitola and Tuukkala) demonstrated significantly higher overall prevalence of haplogroup U (40.9%) than the EMM sites (i.e., Pälkäne, Porvoo, Renko, Turku and Hamina, 18.8%) but also high inter-site variability. Among the EMM samples haplogroup H dominated (U 18.8%, H 46.9%).

This inter-site variability of the haplogroup U/H ratio had a clear spatial pattern also among the IAM samples. The western cluster (IAM south-west: Levänluhta, Luistari and Hollola) had average U and H frequencies of 58.3% and 27.8%, respectively, whereas the corresponding values in the eastern cluster (IAM east: Hiitola, Tuukkala) were 20.0% and 53.3%. In IAM east the highest frequency for an individual subhaplogroup was 30.0% obtained for H1. Strikingly, this U/H ratio is the opposite compared to contemporary eastern and western Finns.

### Differences in haplogroup composition between the sites

Among the 12 Levänluhta samples, five individuals carried haplogroup (hg) U5b, four of which belonged to the sub-hg U5b1b1a. Additionally, the Levänluhta site included three individuals with hg U5a, resulting in a total frequency of 66.7% for hg U5. In contrast, with only two haplotypes of sub-hg H1, the frequency of hg H was well below values observed in modern European populations. The high U5b1b1a frequency resembles that observed today in Saami populations of northern Europe. This actually corresponds well to a related recent study that is showing the close genetic affinity between Levänluhta individuals and modern Saami^18^. However, the Levänluhta individuals also carried mtDNA haplogroups that are absent or rare among the Saami population today, U5a, H1 (0.0–4.0%^34^) and haplogroups K and T. The Levänluhta site clearly showed a unique composition, which resulted in significant genetic distances to all other ancient sites at sequence level, with Φ_*ST*_ values of >10% (see below).

Individuals from the Hollola site, ^14^C dated to 955–1390 calAD (Table 1), also showed a high overall frequency of hg U (64.3%), similar to Levänluhta. However, differences in subhaplogroup distribution between Hollola and Levänluhta suggest a possible non-modern-Saami-like hunter-gatherer ancestry in this region. Interestingly, subhaplogroup U5b1b1a, typical among contemporary Saami, was not observed in Hollola. In contrast, most of the Hollola U haplotypes belong to haplogroups U4 and U5a (frequencies in Hollola 28.6% and 14.3%, respectively), which are rare or absent in Saami today^34^. Moreover, U4 is also rare in modern Finns while the frequency for U5a is around 6%^28,35^. Haplotypes belonging to different subhaplogroups of hg H were more common in Hollola than in Levänluhta, occurring in altogether five samples. Haplogroups K and T were absent in the Hollola sample.

A rather different picture emerged from the Luistari samples, showing a substantial genetic distance to Levänluhta (*Φ*_*ST*_ = 0.134, p < 0.01). Haplogroup U5b1 was entirely absent, and the U haplotypes observed belong to subhaplogroup U4, U5b2 and U2. Lineage U2 is prevalent in some Uralic speaking groups today^36^. The overall haplogroup distribution in Luistari was more similar to the modern European populations dominated by agriculture-associated Neolithic haplogroups H and occurrences of T2 and W1 (see Introduction), than in Levänluhta and Hollola sites.

The two easternmost sites, Hiitola and Tuukkala, proved genetically distant from the western Levänluhta and Hollola sites, despite being approximately contemporaneous with the Hollola individuals. The Neolithic signal in the mtDNA gene pool of ancient Finns in general was much stronger in the east. Both Hiitola and Tuukkala samples showed high frequencies of hg H (61.5% and 47.1%, respectively), together with other Neolithic haplogroups J, K, W and X. Notably, these eastern sites shared three haplogroups: H1a7, H1a8a and H10g. According to GenBank searches these three haplogroups are rare in modern populations: for H1a7 four modern sequences were found, two in Finnish (KY620272 and MF686118), one in Swedish (KJ487971) and one in British (GU797829) populations. For haplogroup H1a8a only two matches were found, one among Finnish (JX153203) and one of an unknown origin (JQ701944), whereas three modern sequences were found for haplogroup H10g: two Finnish (KR732275 and MF497508) and one from Russia (GU122976). Notably, H1* are known to be common in modern Karelia^37^. The eastern sites also comprise rare subhaplogroups U1 (hg U1b2 in Hiitola) and U8 (hg U8b1a2b in Tuukkala), which are atypical for contemporary Finns.

Early modern and modern sites represents similar frequencies of U and H as the combined Iron Age and Medieval East (18.8% and 46.9%, respectively). Contrasting IAM sites and contemporary Finns, EMM sites harbors high prevalence of haplogroup T; frequency in EMM is as high as 21.9%, while in other Finnish populations the frequency is less than 8% **(**Supplementary Tables S5 and S8). Individual JK1954 from Hamina belonged to haplogroup C, which is lacking from contemporary Finns^28^ (Supplementary Table S5) and suggests possible eastern origin. Nevertheless, additional autosomal data is needed to confirm the genetic background of the individual JK1954.

When contrasted with haplogroup frequencies observed in contemporary Finns, our simulations (Supplementary Fig. S2) showed that the ancient sites are significantly different, and that these differences cannot be explained by sampling effects. This applied especially to haplogroup U5 in total and to subhaplogroup U5b in Levänluhta, hg U4 in both Luistari and Hollola as well as hg H1 in the Hiitola dataset.

### Genetic distances among sites and to contemporary Finns

**Figure 3:**
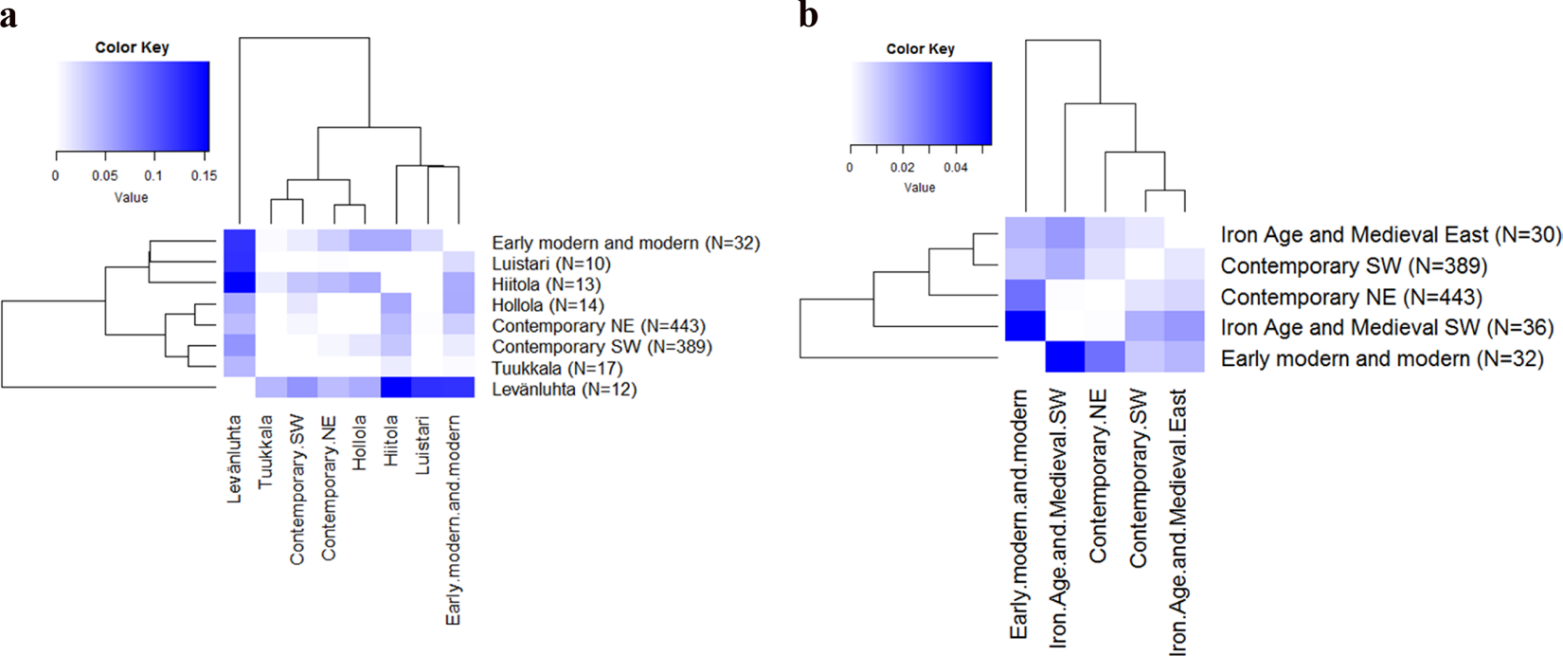
(**a**) Pairwise *Φ*_*ST*_ distances for ancient and contemporary Finns. Early modern and modern Finns consists of individuals from Pälkäne, Porvoo, Renko, Julin and Hamina sites. Contemporary Finns are divided into south-west (SW) and north-east (NE) subpopulations according to^28^. *Φ*_*ST*_ values are presented on a scale starting from zero (*Φ*_*ST*_ values and p-values are presented in Supplementary Table S6). (**b**) Pairwise *Φ*_*ST*_ distances for ancient and contemporary Finns. Iron-Age and medieval (IAM) sites are grouped into subpopulations: IAM south-west consists of Levänluhta, Luistari and Hollola; IAM east consists of Hiitola and Tuukkala. Early modern and modern Finns contain individuals from Pälkäne, Porvoo, Renko, Julin and Hamina sites. Contemporary Finns are divided into south-west (SW) and north-east (NE) subpopulations according to Palo *et al*.^27^ and Neuvonen *et al*.^28^.

When we calculated genetic distances between sites, we observed that Levänluhta differed significantly from all the other sites, except Hollola (*Φ*_*ST*_ = 0.05042, p = 0.02441) and Tuukkala (*Φ*_*ST*_ = 0.04387, p = 0.06055) (Fig. 3a and Supplementary Table S6). The largest distance from Levänluhta was to the eastern Hiitola site (*Φ*_*ST*_ = 0.15468). The distance between Levänluhta and contemporary Finns was smaller but still significant, with a distance to contemporary north-east (NE) *Φ*_*ST*_ = 0.04077 and to contemporary south-west (SW) slightly higher *Φ*_*ST*_ = 0.06473. While Luistari differed only from Levänluhta, the Hollola site differed both from Hiitola and the EMM (*Φ*_*ST*_ = 0.05205 and *Φ*_*ST*_ = 0.05135, respectively, p < 0.05 for both), but not from Levänluhta (*Φ*_*ST*_ = 0.06445, p > 0.05) (Fig. 3a and Supplementary Table S6). Hiitola differed, in addition to Levänluhta and Hollola, from EMM and from both groups of contemporary Finns (*Φ*_*ST*_ = 0.04111 for NE and *Φ*_*ST*_ = 0.03437 for SW). When considering the genetic distances between individual sites, it has to be noted that the relatively low sample sizes might affect the *Φ*_*ST*_ values and the results should be interpreted with caution. However, for pooled IAM and EMM sites (see Fig. 2b), for which the sample sizes are ≥30, the genetic distance calculations should not be that sensitive for bias caused by small sample sizes.

Clustering the IAM sites further roughly according to their geographical location to IAM south-west (hg U more prevalent) and IAM east (hg H more prevalent) further demonstrated the pattern opposite to modern mtDNA diversity distribution (Fig. 3b). IAM south-west differed statistically significantly from contemporary SW (*Φ*_*ST*_ = 0.01670, p = 0.00488) and EMM (*Φ*_*ST*_ = 0.05350, p = 0.00098) but not from contemporary NE (*Φ*_*ST*_ = 0.00036, p = 0.41895). In addition, EMM and contemporary SW differed from each other (*Φ*_*ST*_ = 0.01140, p = 0.04102). Conversely, IAM east differed from the contemporary NE (*Φ*_*ST*_ = 0.00849) more than from contemporary SW (*Φ*_*ST*_ = 0.00514).

Haplotype level median-joining network (Supplementary Fig. S3) demonstrates that ancient and contemporary Finns exhibit in principle same main haplogroups, whereas the most notable differences are within the haplogroup frequencies between the ancient populations. Individuals from IAM eastern sites are more prevalent in the haplogroup H cluster, while individuals from IAM southwestern sites are more concentrated on the haplogroup U cluster. Contemporary Finns are in both clusters, indicating possible mixture of IAM southwestern and IAM eastern populations.

### Main haplogroup frequencies in space and time

To evaluate the possible impact of spatial and temporal factors on the distributions of haplogroup U, largely associated with European hunter-gatherers, and farmer-associated haplogroup H within the IAM sites, we performed multinomial logistic regression analyses. In a stepwise forward analysis, the only statistically significant independent variable explaining the differences in the haplogroup composition was the distance from eastern reference point Lahdenpohja (compared to ‘H’ and ‘Others’ significance for Lahdenpohja was 0.013 and 0.103, respectively) (Supplementary Table S7). Neither the ages of the samples nor distance from the southern and western reference points were requisite for the best-fit model. However, the addition of the eastern reference point significantly improved the fit between model and data (p = 0.027). Based on the odds ratios, it is less likely that an individual from southwest belongs to haplogroups ‘H’ or ‘Others’ than an individual from an eastern archaeological site. Similar results were obtained when using hunter-gatherer associated haplogroups (U and V), farmer associated haplogroups (H, J, K and T) and ‘Others’ as categorically distributed dependent variables. We chose to include the haplogroup V as ‘hunter-gatherer’ while there is no direct evidence for association of hg V with the hunter-gatherers. This is assumed here because of V’s northern distribution and its high prevalence (up to 58%^34^) among the Saami, the archetypal nomadic population lacking many farmer-associated haplogroups^34,38^. Distance from the eastern reference point was the only predictor included in the model (with significance of 0.031 for farmer associated haplogroups and 0.082 for other haplogroups). Assuming that haplogroups U and H can be associated to hunter-gatherers and farmers, respectively, the results suggest a spread of the more central European like, farmer-related haplogroups spreading from the east. However, as mentioned above, association of hg V is unclear. Omitting V from the hunter-gatherer group does not change results noteworthily (Supplementary Table S7).

### Genetic affinities of ancient Finns to other ancient and contemporary populations

**Figure 4:**
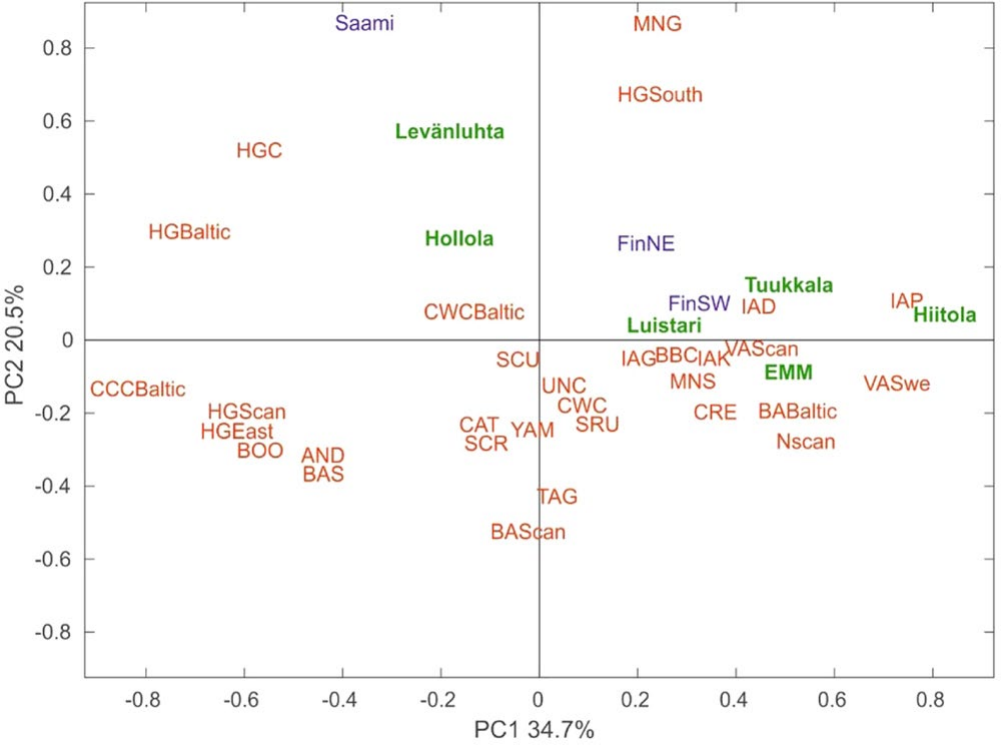
PCA biplot based on mitochondrial haplogroup frequencies. Ancient populations presented in this study (marked with green): Levänluhta, Luistari, Hollola, Hiitola, Tuukkala and Early modern and modern Finns (EMM). Contemporary populations (marked with blue): north-east Finland (FinNE), south-west Finland (FinSW), Saami (SAA). Ancient populations (marked with red): Andronovo culture (AND), Baltic Bronze-Age (BAB), Siberian Bronze-Age (BASi), Scandinavian Bronze-Age (BASc), Bell Beaker culture (BBC), Siberian Early Metal Period (BOO), Catacomb culture (CAT), Comb ceramic culture Baltic (CCCB), Crete Minoans (CRE), Corded Ware culture (CWC), Baltic Corded Ware Culture (CWCB), Baltic hunter-gatherers (HGB), central European hunter-gatherers (HGC), eastern hunter-gatherers (HGE), Scandinavian hunter-gatherers (HGSc), south European hunter-gatherers (HGSo), Denmark Iron-Age (IAD), Germany Iron-Age (IAG), Kazakhstan Iron-Age (IAK), Poland Iron-Age (IAP), Germany Middle-Neolithic (MNG), southern Europe Middle-Neolithic (MNS), Scandinavian Neolithic (NSc), Scythians from Russia (SCR), Scythians from Moldova and Ukraine (SCU), Srubnaya culture (SRU), Tagar culture (TAG), Unetice culture (UNC), Scandinavian Viking-Age (VASc), Sweden Viking-Age (VASw),Yamnaya culture (YAM). The contribution of main mitochondrial haplogroups are represented by loading vectors in Supplementary Fig. S4.

To further explore the affinity of ancient Finns to other ancient and contemporary populations, we carried out principal component analysis (PCA) based on haplogroup frequencies. We plotted the first two components of the PCA plot for ancient Finns, 31 other ancient populations, contemporary Finns and Saami, which account for 55% of the total variance (Figs 4, S4). Interestingly, southwestern Iron-Age sites Levänluhta and Hollola fall close to hunter-gatherer populations from Baltic, Central and Southern Europe. In addition Levänluhta is located in proximity to modern day Saami. This suggests the hunter-gatherer type of maternal ancestry in these two sites. In contrast, eastern IAM sites Hiitola and Tuukkala, EMM sites and contemporary SW Finns clustered with European Neolithic, Bronze-Age and Iron-Age populations. The southwestern site Luistari, as well as the contemporary NE Finns, were located roughly between these two clusters, indicating a possible mix of maternal ancestry from hunter-gatherers and Neolithic farmers. However, as with the genetic distances presented in Section 2.6., the small sample sizes of ancient populations might distort the haplogroup frequencies to deviate from the original source population, subsequently affecting PCA. To evaluate the possible bias, we performed random subsampling of contemporary SW and NE Finns (fifty iterations, for each N = 15) and carried out PCA with the same reference populations as for Fig. 4. Supplementary Fig. S5 demonstrates the amount of variation induced.

## Discussion

Here we report 103 human mitochondrial DNA genomes from approx. 300 AD to 1800 AD, a transect both in time and space, which represents thus far the largest collection of individuals with ancient human DNA analyzed from Finland. Analysis of the prehistoric samples from Iron-Age and medieval sites from western and eastern Finland revealed a high overall prevalence of haplogroup U in southwestern sites, in stark contrast with a high frequency of haplogroup H in the east, which is opposite to what is observed in modern day Finland. Moreover, there is relatively high differentiation between the ancient sites.

### Genetic layers of mitochondrial variation among the Iron Age and Medieval Finns

Unexpectedly high variation in maternal lineages could be observed between the southwestern Levänluhta, Luistari and Hollola Iron-Age sites. Especially the distribution of U subhaplogroups differed clearly between sites: The oldest site, Levänluhta, represented a high frequency of U5a and the modern Saami-related haplogroup U5b1b1a, which is present in contemporary Finns only in moderate frequency of around 3.0%^35^. Indeed, recent studies considering Levänluhta, in which nuclear genomes have been retrieved, confirm the genetic continuation with the modern Saami population^18,19^. The strong drift experienced by the Saami groups of present day, shown by their high levels of LD throughout genome and low diversity in uniparental markers (See^38^ and references therein) could explain why some mtDNA lineages, such as U5a, would have vanished from present day Saami.

Both Luistari and Hollola lacked the U5b1b1a, but instead Hollola displayed a wider variety of other U5 subhaplogroups, such as U5a1, U5a2 and U5b2. In addition, Luistari and Hollola sites showed relatively high frequencies of different subhaplogroups of U4 (i.e., U4a, U4b and U4d), which are rare in contemporary Finns and absent from modern Saami. Instead, in contemporary populations, U4 exists in high frequencies in Volga-Ural region (up to 24% in Komi-Zyryans)^36^ and with lower frequencies around the Baltic Sea, such as in Latvians and Tver Karelians (both around 8%)^37^. Taking into account that U4 have been prevalent in neighboring areas among Scandinavian^10,39–43^ and Baltic hunter-gatherers^12,13,44^, Baltic Comb Ceramics Culture^12–14^ and in Siberia during the Early metal period^11^, we might be observing ancestries belonging to an earlier layer of ancient inhabitants of the region.

Taking these different distributions of mtDNA haplogroups from the Iron-Age and medieval sites into consideration, our results suggest three different streams of mitochondrial ancestry: Saami-like haplogroups (U5b1b1a, possible also U5a), non-Saami-like hunter-gatherer related haplogroups (especially U4) and haplogroups associated with Neolithic farmers (H, J, K and T). In this context we use ‘Saami-like’ as a term that shows genetic continuity with modern-day Saami groups. Different proportions of these ancestries could be observed both in later EMM sites and also modern-day southwestern and northeastern Finns. This suggests a fluctuation of each of these mitochondrial ancestry proportions over space and time.

### The ancient distribution of mtDNA lineages contradicts the contemporary east-west divergence

The Finnish population has been a subject of multitude medical genetic studies for many decades. Assessments of genetic diversity have revealed a number of idiosyncrasies in the modern Finnish gene pool. These include, for instance, the enrichment of c. 40 rare genetic diseases and the absence of some major ones in the rest of European metapopulation, as a clear distinction from the largely clinal differences observed in most of Europe^45^. Furthermore, these studies have demonstrated the existence of notable genetic differentiation between southwestern and northeastern parts of Finland^24,26,27^. This differentiation is especially pronounced in Y-chromosomes, showing opposite frequency trends of haplogroups N1c (25% SW, 75%NE) and I (56% SW, 24% NE)^26^.

The modern mitochondrial DNA diversity in Finland resembles that observed in the Central Europe, but holds a relatively high overall frequency of haplogroup U, and also a notable proportion of subhaplogroups which have frequency peaks in or are exclusive to Finland^35^. The genetic substructure within Finland is minimal when at the level of mtDNA haplotypes are considered, but pronounced in the frequencies of haplogroups assumed Paleolithic (here U and V) or Neolithic (H, J, K, T) in Europe: the palaeolithic haplogroups are more common in the north-east (“Contemporary NE” subpopulation), and Neolithic haplogroups in the south-west (“Contemporary SW”). This, together with the Y-chromosomal subdivision, has been interpreted to reflect an ancient border between populations relying on farming (south-west Finland) and foraging (north-east). The observed genetic border running diagonally from north-west to south-east coincides with differences in a number of linguistic and cultural differences all the way to folk traditions. It also coincides with the first medieval political border, the Treaty of Nöteborg, between the Swedish and Novgorodian spheres of influence agreed in 1323 AD (see^28^).

The ancient mitochondrial genomes analyzed here show a notable pattern opposite to the modern variation: mtDNA types usually associated with the hunter-gatherer communities were significantly more common in the ancient western cluster (Levänluhta, Luistari and Hollola) than in the east (Hiitola, Tuukkala), with the haplogroup U frequency as high as 58.3%. In contrast, the farming-related lineages were observed in particular in the ancient eastern cluster. This pattern of division between the ancient sites, and the contradictions with their respective local modern population frequencies emerged also in formal testing of pairwise *Φ*_*ST*_ values: the western cluster was closer to the modern NE subpopulation than to the modern SW subpopulation whereas the eastern cluster showed closer affinity with the modern mtDNA variation in southwestern Finland.

### Bidirectional expansion of agriculturally oriented populations into Finland?

Assuming that the haplogroup composition has correlated with the mode of subsistence, the observed pattern of east to west transect suggests a bidirectional spreading of agricultural human groups into Finland. Although there is evidence of sporadic small-scale cultivation in southeastern Finland already during the Neolithic Stone Age (*c*. 5300–4000 BC)^46,47^, the start of agriculture in Finland has been traditionally associated with the Corded-Ware Culture (CWC) arriving across the Baltic Sea approximately 4700 years ago. Indeed, there are scattered findings of animal husbandry from southwestern parts of country starting from *c*. 2500 calBC^48^, but in general archaeological evidence supporting transition to agriculture as a consequence of introduction of Corded-Ware culture, are sparse (for discussion see^49^). Some independent observations of animal domestication^50^ and cultivation (see^22^ and references there in) are identifiable during the Bronze Age, but documentation remains still very limited. This suggests that cultivation has probably been relatively uncommon and local for centuries, as little direct evidence for cereal cultivation in Finland prior Iron Age exists^22,49^. Pollen records show notable increase of cereals starting only at 100 AD and reaching maximum as late as 1300 AD^22^ overlapping the time span of Iron Age and Medieval sites presented in this study.

As a support for the late introduction of farming populations in to Finland we do not see strong affinities of western IAM to for example the CWC maternal gene pools from Estonia and Lithuania^6,12–14^, suggesting either that the mtDNA gene flow between these two regions has been low or that shared mtDNA variation had dissolved before the Iron-Age in Finland. Alternatively, the CWC expansion may have been largely male-driven as suggested by^51^. However, we observe a strong Neolithic signal in the Iron-Age mtDNA pool in Eastern Finland, thus rather suggesting a southeastern/eastern arrival route of an agro-pastoralist population into the country. Interestingly, their maternal genetic legacy also corresponded to the contemporary modern Finland, especially in SW. We therefore propose that either there has been east-to-west directed gene flow during the Middle Ages, after the introduction of agricultural haplogroups into the east, or that the late change in SW maternal gene pool may reflect recent immigration from more western/southern sources, such as the migration from Sweden during the Swedish reign in Finland (from 1200s–1809). Iron-Age has evidenced high mobility around the Baltic Sea, as evidenced by the genetic and isotope analyses of human remains from 10th to 12th century in Sigtuna, eastern Sweden^52^.

The multinomial logistic regression analysis lent support to the eastern introduction of agriculturally related maternal ancestry. The likely migration routes for the observed ancestral elements were investigated through different combinations of factor dependencies as the multinomial logistic regression. The test revealed the distance from Lahdenpohja on the eastern border of Finland as the only statistically significant variable explaining the differences in the haplogroup composition. Neither time scale, nor distance from the southwestern locations (i.e. Hanko and Uusikaupunki) were supported by the best-fit model. It thus seems likely that the major spread of haplogroup H can be explained by presuming its introduction via the eastern landroute. In accordance with the inference here, population genetic studies of many organisms in Finland as well as in all Fennoscandia have suggested bidirectional colonization of the current habitats. The reasons behind this are largely geographical: the Baltic Sea acts, for most species, as a migration barrier.

The Neolithic farmer-related signal in the mtDNA diversity in the Iron-Age samples is mainly found in the southeast, whereas in contemporary population it predominates in the southwest. The reasons for this discrepancy are likely diverse, and could be affected by such recent events as the evacuation of nearly 0.5 M inhabitants of Karelia during the World War II and their resettling into the area of current Finland. However, these evacuees were resettled rather evenly across southern Finland and should not create the observed pattern. It rather suggests that the division between SW and NE Finland had still been more substantial in the early 1900s. Another, more fundamental explanation for the genetic subdivision comes from the environmental demands of sedentary farming. In southwestern Finland the soil is more amenable to field-farming and due to the warming Atlantic effect that gradually shades into more arid continental climate, the growing season in Finland is the longest in the southwestern coast. These environmental differences follow the NW-SW border similarly to the genetic distances. As the country has been sparsely inhabited until modern times, it is plausible that farming oriented populations, in search of more favorable conditions, have over the centuries concentrated into the SW parts of the country.

The mitochondrial DNA genomes from Iron-Age Finland show variation that can be linked to either hunter-gatherer or agricultural human groups. These elements are still present in the mitochondrial gene pool of contemporary Finns but relatively evenly distributed throughout the country. In contrast, the Iron-Age mtDNA variation show significant differences between sampling sites, with hunter-gatherer and farmer-associated elements dominating in different regions than today. Rather surprisingly, the agricultural population signal has been stronger in eastern Finland in the past, which might reflect a bidirectional arrival of farming-associated populations into Finland.

## Materials and Methods

### Sample selection

The human skeletal remains used in this study were collected from five archaeological sites and five historical cemeteries (for more detailed information of the sites and references for original publications in Supplementary Material S1). Archaeological sites include Levänluhta, used as a burial place from Roman Iron Age until the end of Merovingian Period (archaeological dating 300–800 AD), Luistari, consisting of graves from Merovingian to Crusade Period (archaeological dating 600–1200 AD) and Hollola, Hiitola and Tuukkala, largely Christian-style cemeteries spanning from Crusade Period to Early Middle Ages (archaeological datings 1050–1400 AD, 1200–1500 AD and 1200–1400 AD, respectively) (Fig. 1, Table 1 and Supplementary Material S1). The five historical sites include the cemetery of the Church of St. Michael in Pälkäne, the cemetery of the Church of St. Jacob in Renko, the Cathedral site in Porvoo, the Julin’s site in Turku, and the Ryazan regimental church cemetery in Hamina. Samples were obtained from the archaeological collections of the Finnish Heritage Agency, Department of Archaeology in the University of Turku, Department of Anatomy in the University of Helsinki, and the Peter the Great Museum of Anthropology and Ethnography (Kunstkamera), Russian Academy of Sciences.

Contextual archaeological evidence, such as grave goods and burial customs, together with radiocarbon analyses were used to confirm the dating of each site and/or individual. Details of dating, sample sizes and number of genomes obtained are presented in Table 1. Detailed information on individual burial sites and samples are given in Supplementary Material S1 and Supplementary Table S1.

### Reference populations used in comparative analyses

To evaluate possible changes in the Finnish mitochondrial gene pool during the past thousand years, samples were compared to HVR1 + HVR2 (16024–16385, 72–340) data from 832 modern Finns^27^ for which the county-level geographical origin is known. In order to compare the mitochondrial profile of ancient Finns to other ancient nearby populations, haplogroup frequencies were collected from 31 ancient populations (Supplementary Table S8).

### Sampling

All samples were processed in dedicated aDNA facilities with regularly UV:d and bleach-treated laminar hoods, inside a clean room space. For Levänluhta, Porvoo, Renko, Pälkäne and Hamina sites, sampling was conducted at the University of Tübingen, Germany from the start. For the Luistari, Hiitola, Hollola and Tuukkala sites, the bone powder was produced in a clean-room space for small-scale ancient DNA work at the Helsinki University Department of Forensic Medicine, and then transferred to Tübingen University facilities, stored in plastic tubes. All subsequent laboratory work with the bone powder was conducted at the facilities in Tübingen. A protective overall, facemask, hair net and two layers of disposable gloves were used at all times when handling the samples. Decontamination was carried out by ultraviolet light exposure of the plastic ware, reagents and samples, and by removal of the immediate surface at the point of sampling before drilling into the bone. Teeth were sawed in two, with the exception of the Levänluhta samples, where dentine was already exposed due to heavy fragmentation. Dental pulp and the surrounding dentine were used for DNA sampling, drilling into the crown or the root. For the petrous part of the temporal bone, a wedge was cut off to reveal the inner ear channels and sample was taken from the inside of the channel, as previously described^53^. A dentist’s drill together with cooled-down drill heads for minimal heat exposure were used at the University of Tübingen facility and a “field kit” with Dremel or dentist’s drill were used at the Helsinki University Department of Forensic Medicine.

### Extraction of ancient DNA

The extraction was performed according to a modified version of the original protocol^29^. For each sample, ~50 mg of bone powder was used for the extraction by eluting it in 100 μl of TET (10 mM Tris-HCL, 1 mM EDTA pH 8.0, 0.1% Tween20). The extracts were used to prepare DNA libraries of 20 μl, without additional treatments. To enable multiplex sequencing, the double-stranded library preparation and the subsequent indexing procedure were performed according to standard recommended protocols for ancient DNA^54,55^. The original molecular copy number in the DNA library, as well as the subsequent indexing efficiency, were measured by qPCR, using AccuPrime Pfx polymerase. The molecular copy numbers in pre-indexed libraries varied, ranging from ~1–100 × 108 copies/μl, and the indexed libraries from ~1–100 × 1011 copies/μl, indicating a successful library composition and admissible indexing efficiency. The indexed libraries were amplified using PCR, with heating cycles chosen individually per library according to the copy number after indexing. Amplified libraries were purified using MinElute spin columns with the standard protocol provided by the manufacturer (Qiagen). A qPCR together with Agilent Bioanalyzer 2100 device, and a DNA1000LabChip were subsequently used on the amplified libraries to measure the concentration of DNA, as well as fragment size distribution. A positive control extracted from a cave bear bone, to confirm the success of extraction and library preparation, as well as two negative laboratory controls to measure the levels of contamination were carried along for every batch of 10–16 samples.

### Mitochondrial capture and sequencing

Mitochondrial genomes were achieved using a mitochondrial in-solution capture as described in Maricic *et al*.^56^. Complete human mitochondrial DNA sequence was used to produce in-house made baits, which were ligated to adapters. The bait DNA was then purified and denatured to the single stranded form, and attached to streptavidin-coated magnetic beads. Pools of 4–6 samples, combined in equal mass ratios for altogether 2 μg of DNA, were captured with the above mentioned beads, and sequenced on the Illumina platforms: for samples from sites Turku, Hiitola, Tuukkala and Hollola, along with four samples from the Luistari site (TU619, TU621, TU622, TU623), single-end sequencing data was produced on HiSeq4000 run for 75 + 8 + 8 cycles, whereas samples from all the other sites, including the rest of the samples from Luistari, paired-end data was produced with NextSeq500 for 2 × 150 + 8 + 8 cycles, at the Max Planck Institute for the Science of Human History, Jena.

### Processing of the sequence data

Raw-read sequencing data were processed using the EAGER-pipeline for aDNA sequencing data^57^. Reads were demultiplexed using both indices, and the adapters were clipped off with the AdapterRemoval program integrated in EAGER, with minimum overlap set to 1 bp. Short reads reaching a minimum of 10 bp overlap were merged, validated by the paired-end fragment compatibility. For the single-end-sequenced samples this step was omitted. Minimum read length of 30 bp and base quality of 20 was required for all final reads. The reads were aligned to the complete human reference genome Hg19 with the BWA mapping algorithm for the shotgun sequence data, whereas CircularMapper, a custom-made tool for circular genomes included in EAGER, was used to map the enriched mitochondrial reads to the human mitochondrial reference genome (revised Cambridge reference sequence, rCRS^58^).

### Authentication of ancient DNA and haplogroup assignments

In order to evaluate the authenticity of the reconstructed mitochondrial genome sequences, contamination rates, read-length distributions and deamination patterns at the 5′ and 3′ ends of DNA fragments were inspected for each sample with the program MapDamage^59^ integrated in EAGER. Further contamination estimation was carried out with Schmutzi^32^ and ContamMix^33^ programs.

The mitochondrial consensus-calling in Schmutzi was used to produce the consensus sequences of both the endogenous source and the most likely single contaminating source. Complete consensus sequences were called against the rCRS with a filter value of q20. For the Schmutzi-based consensus sequences a manual correction was performed for position 3107 to correspond to the “N” embedded in rCRS. The mtDNA haplogroups were determined using HaploGrep2^60^ with respect to PhyloTree version 17^61^.

For contamination estimation in ContamMix, consensus sequences created by Schmutzi were combined with a reference dataset of 311 mitochondrial genomes from worldwide populations (provided by ContamMix) and aligned with mafft version 7.305^62,63^. The untrimmed mitochondrial reads from the Eager pipeline were then extracted from BAM files into fastQ files and mapped back to the assembly. ContamMix then evaluates whether the reads assign more probably to their respective consensus or one of the worldwide mitochondrial genomes, i.e possible contaminant source. The ContamMix was run with trimming of seven bases of each side of the read to remove the accumulated damage typical for ancient DNA.

The mitochondrial genome sequences with highest ContamMix estimates were further visually inspected in Geneious 11.0.3 (www.geneious.com). In this inspection, the majority call support for relevant diagnostic mutations against the rCRS reference genome was compared to the PhyloTree version 17. We applied the automated variant caller in Geneious to the alignments with minimum support of 3x coverage and variant frequency of 66.6% for diagnostic SNPs to confirm the authenticity of the haplogroup assignments.

### Radiocarbon dating

Radiocarbon dates were produced by the Laboratory of Chronology, Finnish Museum of Natural History Luomus in Helsinki, Finland (Hela) and Klaus-Tschira C14-laboratory in Mannheim, Germany (MAMS). Bone collagen was extracted with the modified Longin method^64,65^, the collagen samples combusted, graphitized and measured by using Accelerator Mass Spectrometry (AMS). The results are provided as conventional radiocarbon dates without potential reservoir effect corrections. The radiocarbon dates were calibrated using the OxCal program version 4.3^66^, IntCal 13 as the calibration curve^67^. For sites with four or more individuals with ^14^C dates also the boundaries for phase’s start and end were determined with OxCal 4.3. Example of the OxCal code is given in Supplementary Material S2. Timescale discussed throughout the text is defined as calendar years.

### Statistical analyses

A summary of statistical analyses performed in this study for different sets of populations and dataset (i.e., complete sequence, HVR1 + HVR2, haplogroup frequencies) is presented in Table 2.

**Table 2:** Overview of statistical analyses and datasets used in this study.

Analysis	Data type	Populations used	Results presented in
Basic diversity indices	Complete sequences	Ancient Finns	Supplementary Table S4
*Φ*_ST_	HVR1 + HVR2	Ancient and contemporary Finns	Fig. 3 and Supplementary Table S6
Multinomial logistic regression	Presence/absence of haplogroup	Ancient Finns	Supplementary Table S7
Principal component analysis (PCA)	Haplogroup frequencies	Ancient Finns, other ancient and contemporary populations (Supplementary Table S8)	Figs 4 and S4
Evaluation of the possible sampling bias in the observed haplogroup frequencies	Haplogroup frequencies	Ancient and contemporary Finns	Supplementary Fig. S2
Network analysis	HVR1 + HVR2 sequence data	Ancient and contemporary Finns	Supplementary Fig. S3
Evaluation the impact of small sample size on PCA	Haplogroup frequencies	Contemporary Finns, ancient Finns and other ancient populations (reference populations presented in Supplementary Table S8)	Supplementary Fig. S5

For the statistical analysis, sequences were aligned with Muscle v3.8.31^68^. To explore the genetic diversity within ancient populations, basic diversity indices such as haplotype diversity, mean pairwise distance and nucleotide diversity were calculated for the complete mitochondrial genomes with Arlequin 3.5.2.2^69^. To determine genetic distances between ancient and modern Finns, pairwise *Φ*_*ST*_ values based on the sequence data were calculated with Arlequin 3.5.2.2. Genetic distances, were calculated by utilizing HVR1 + HVR2, as the contemporary reference data with detailed geographical origin were restricted to HVR regions only^27,28^. To estimate the significance for the *Φ*_*ST*_ values, permutation tests with 10000 permutations were used. The visualization of the *Φ*_*ST*_ values was done by R heatmap.2 function with hierarchical clustering based on the Euclidean distance. The best-fit models for different datasets were estimated with jModelTest^70^. The substitution model used was Tamura & Nei^71^ with gamma correction (shape parameter α = 0.67) and Tamura & Nei with gamma correction (*α* = 0.44) for the complete sequence data and HVR1 + HVR2 data, respectively. Poly-C region (positions 309–315), AC indels (positions 515–522) and mutational hotspot at position 16519 were masked for the population level analysis. Further to evaluate the relation of ancient individuals to contemporary Finns on a haplotype level, median-joining network analysis^72^ was performed with PopArt^73^. Positions bearing more than 5% of missing data were masked for the network analysis.

To statistically test the impact of possible factors affecting the spatial and temporal distribution of haplogroups U and H in our ancient samples, we conducted a multinomial logistic regression analysis. Each individual was considered as separate occurrence and haplogroups ‘U’, ‘H’ and ‘Others’ were set as categorical dependent variables. Because the main interest was to estimate the impact of time and geography on the occurrence of the haplogroups, the following independent variables were chosen: (1) Median age of sample (or the mean age of the site if radiocarbon dates were not available) (2) site’s distance (in km) from southern reference point Hanko, (3) site’s distance (in km) from western reference point Uusikaupunki and (4) site’s distance (in km) from eastern reference point Lahdenpohja (see Fig. 1). These geographical points were chosen to represent the most plausible entry points of different migration routes to the study area, south (Hanko on the southern coast), west (Uusikaupunki on the western coast) and east/south-east (Lahdenpohja). The last mentioned represents the eastern migration route both along the Karelian Isthmus and north of Lake Ladoga, strongly supported by archaeological evidence^21^. For multinomial logistic regression a stepwise forward method was used with entry probability 0.05, and probability was tested with likelihood ratios. To evaluate the impact of grouping the haplogroups into hunter-gatherer and farmer related hgs’ also categories ‘U + V’, ‘H + J + K + T’ and ‘Others’ were tested. In addition, categories ‘U’, ‘H + J + K + T’ and ‘Others’ were tested, due to controversial definition of haplogroup V (See Section 2.7). Analyses were performed with IBM SPSS version 25 (IBM Corp. Released 2017, IBM SPSS Statistics for Windows, Version 25.0 Armonk, NY: IBM Corp.).

To trace genetic affinities between ancient Finns and other ancient populations, we visualized haplogroup composition of each site (based on haplogroup frequencies) using principal component analysis (PCA). PCA was computed using MATLAB and Statistical Toolbox Release 2015b (The MathWorks, Inc., Natick, Massachusetts, United States). Populations and haplogroup frequencies used are presented in Supplementary Table S8.

## Supplementary information

Supplementary Materials and Figures

Supplementary Tables

## Data Availability

Complete mitochondrial sequences will be deposited in GenBank under accession numbers MN540463-MN540565.
