# Supplementary material for: Human mitochondrial DNA lineages in Iron-Age Fennoscandia suggest incipient admixture and eastern introduction of farming-related maternal ancestry
Source: Sci Rep. 2019 Nov 15;9:16883. doi: 10.1038/s41598-019-51045-8 (PMC6858343; doi:10.1038/s41598-019-51045-8)
Supplement: Supplementary file 1 — Supplementary Materials and Figures [file 41598_2019_51045_MOESM1_ESM.pdf]

Human mitochondrial DNA lineages in Iron-Age Fennoscandia suggest incipient admixture and eastern introduction of farming-related maternal ancestry

Supplementary Materials and Figures

Sanni Översti[1,†,*], Kerttu Majander[1,2,3,†], Elina Salmela[1,2], Kati Salo[4], Laura Arppe[5], Stanislav Belskiy[6], Heli Etu-Sihvola[5], Ville Laakso[7], Esa Mikkola[8], Saskia Pfrengle[3], Mikko Putkonen[9], Jussi-Pekka Taavitsainen[7], Katja Vuoristo[8], Anna Wessman[4,7], Antti Sajantila[9], Markku Oinonen[5], Wolfgang Haak[2], Verena J. Schuenemann[3,10], Johannes Krause[2,‡], Jukka U. Palo[9,11,‡], Päivi Onkamo[1,12,‡]

Material S1. Detailed information about archaeological sites and samples.

Material S2. Example of the OxCal code used to determine the start and end distribution.

Figure S1. Phase boundary outputs for each site containing four or more ^{14}C dates.

Figure S2. Evaluation of the possible sampling bias in the observed haplogroup frequencies.

Figure S3. Median-joining Network for ancient and contemporary Finns.

Figure S4. PCA with main loading vectors.

Figure S5. The impact of small sample size on PCA.

(**Supplementary Tables S1-S8** are presented in separate Excel file)

Material S1. Detailed information about archaeological sites and samples.

Two of the archaeological sites, Levänluhta and Luistari, represent the oldest burial sites with preserved unburnt human skeletal remains in Finland. Both sites have been the target of extensive archaeological studies. Levänluhta, a lake burial in Isokyrö, southern Ostrobothnia, has yielded 98 macroscopically well-preserved skeletons in several excavations conducted over the last 200 years. The grave goods from Levänluhta have stylistic features from around the Baltic Sea region but the burial cannot be linked to any known settlement or more distinct archaeological findings. A total of 13 tooth samples from Levänluhta individuals were included in this study (**Table 1**).

The Luistari site in Eura is a large burial ground consisting of 400 excavated graves from the Merovingian, Viking and Crusade Periods in Finland. A total of 25 individuals were included from Luistari, of which five were assigned to the Merovingian Period, 14 to the Viking Period, and the remaining six roughly to the Crusade Period, based on artefact datings. The data was further complemented by another Crusade Period burial ground of Kirkkailanmäki in Hollola, contributing 20 individuals. Two more eastern burial grounds, Kylälahti Kalmistonmäki in Hiitola (today Khiytola in Russian Karelia) and Tuukkala in Mikkeli, dating from the Crusade to medieval Periods, provided 18 and 30 individuals, respectively.

The remains from the Early-Modern period individuals originate from five Christian churchyards: four individuals from Pälkäne (cemetery of the Church of St. Michael), where the earliest burials date to the 13th century and the latest ones to the 19th century; eight individuals from Renko (Church of St. Jacob) and nine from Porvoo (Cathedral site) dated based on the archaeological context to14th - 18th century; five from Turku (Julin's site) dated to 16th - 17th century; nine from Hamina (Ryazan regimental church cemetery) dated to 18th century.

Levänluhta

Levänluhta cemetery in Isokyrö, historical province of Osthrobotnia is an exceptional middle Iron-Age (4th to 7th century AD) burial site, because here the burials have been done in water. The prevailing form of burial in Finland during this time, until the beginning of the Crusade Period (mid-11th century) was cremation. Levänluhta was probably a pond or a lake during its time of usage. It seems that these individuals were brought to a remote area, outside the local Iron-Age settlement, to be buried in a very different way than was the norm [1-3].

Luistari

Luistari site in Eura in south-west Finland includes a Bronze-Age settlement site, Iron-Age cremation burial cairns and a medieval inhumation cemetery. Inhumating began during the Merovingian Period (7th century AD) and continued at least until the 14th century AD [4, 5]. The site has been extensively studied, yielding c. 400 graves dating to Merovingian and Viking Periods and over 800 later, but very few of these have human skeletal remains [5-7].

Hollola, Kirkkailanmäki

Kirkkailanmäki cemetery in Hollola, historical province of Häme, is dated from 11th to 14th centuries [8-10]. The cemetery has furnished and unfurnished inhumations as well as cremations [8, 11]. The grave finds are from Crusade Period and early medieval times and they show influences from several different origins: Eastern influences in the site are shown by Karelian type of brooches [8, 9] and a Russian type of pendant [8, 9, 12]. Southern influences in the site are shown by an Estonian type of pendant [8, 13] and brooches originating from the southern side of the Baltic Sea [8, 10, 14]. A bird pendant found from one grave is of western Finnish type, but it originates from Livonia in the modern Latvia [8, 14].

Hiitola, Kylälahti Kalmistonmäki

Kylälahti Kalmistomäki cemetery in Hiitola in the north-western bank of Ladoga Lake shore is in modern day Russia and dates to Crusade Period and medieval times [15]. Field investigations were carried out in 2006–2009 on the Kalmistomäki Hill by an archaeological expedition of the Museum of Anthropology and Ethnography (Kunstkamera) Russian Academy of Sciences (Saint-Petersburg) in cooperation with the University of Turku (Finland). The total excavated area amounted to 426 m2. The number of undisturbed burials uncovered was 93, of which 91 are inhumations and two cremations. The number of excavated burials containing grave goods (n=51) is the largest among all the known cemeteries, including the burial grounds in present-day eastern Finland and Karelia (see [15]).

Tuukkala

Tuukkala cemetery in Mikkeli is dated from 13th to 15th centuries and it contains both inhumations and cremation burials. With over 80 inhumation burials, this eastern Finnish burial site is the largest and richest inhumation cemetery excavated in the area of the historical Savo province [16, 17]. Tuukkala cemetery was found and mainly excavated in 1886, and several excavations have been conducted since then (see [17]). Despite the extensive excavations, no signs of a church have been found and there are arguments for and against the cemetery being Christian [16, 18]. Osteological research has showed signs of severe trauma and exceptionally tall men [11, 17, 18]. On the basis of the grave goods taller men could have a western origin and the shorter men an eastern origin [18].

Pälkäne, Church of St. Michael

The medieval stone Church of St. Michael in Pälkäne has been built around the year 1500, but based on the radiocarbon datings the burial ground has been used already from 13th century [19, 20]. During the year 2003 over fifty burials were excavated [19]. Most of these burials were unfurnished, and based on the archaeological context they are dated to 17th and 18th centuries [19]. Osteological analysis revealed that the skeletons show shorter stature than other post-medieval sites in Finland [11].

Porvoo, Cathedral site

The first wooden church at the Porvoo Cathedral site was built in the middle of 13th century, followed by a stone church built in the beginning of the 15th century [21]. The cathedral's burial ground dates from 14th to 18th centuries although the individuals used in this study date most likely to 17th and 18th centuries based on the burial customs (see [11, 22]). Porvoo is situated along the southern coast of Finland and there have been trade connections and even migration to Porvoo from all over the Baltic Sea, especially from Tallinn and Stockholm [11, 23, 24].

Renko, the Church of St. Jacob

The stone church of St. Jacob in Renko has been built in the beginning of 16th century, although based on the coins found in the excavations, it has been proposed that site had a wooden church already in the beginning of the 15th century [25]. In the middle of 17th century the stone church was abandoned, but it was renovated and reintroduced at the end of 18th century [21]. In the excavations during 1984 around 70 burials were identified [25]. The most famous historical road, Hämeen Härkätie from Turku to Hämeenlinna passes by Renko and historical records demonstrates some migration events at the end of 18th century [26]. The origin of the immigrants in the parish is not known, but most likely most came from neighboring provinces.

Turku, Julin's site

Julin's site consisted in addition to the Church of the Holy Spirit and its cemetery also a hospital and/or a house of the poor people. Based on its location, it has been assessed that the cemetery, dated to 16th and 17th centuries [27], was a cemetery for the lower social class. Several excavations have been conducted and several hundreds of individual burials have been identified (See [11]). Julin's site lies in Turku, which is the first town in Finland (established at the turn of 13th and 14th centuries). The population density in Turku has been higher than any other sites in this research even in the following century and the city has also had most vivid trade and other contacts across the Baltic Sea.

Hamina, Ryazan Regimental Church

Ryazan Regimental church in Hamina, on the southeastern coast of Finland, was presumably built by the Russian army ca. 1744 [28]. Excavations conducted in this Orthodox cemetery in 2011 found traces of 35 individual burials [28]. The cemetery has an exceptional demographic profile with 4 young adult males, likely soldiers, and rest of the deceased were children less than 7 years old [11, 28]. Based on this bias it has been proposed that the most probable cause of death for individuals buried in the Ryazan Regimental Church's burial ground was infectious disease(s) [28].

Material S2. Example of the OxCal code used to determine the start and end distribution.

Example is presented for Luistari site, for which all ten individuals included in this study were radiocarbon dated.

```
Sequence("Luistari")
{
Boundary("Start of Luistari");
Phase("Luistari")
 {
R_Date("Luistari TU620  Hela-3962", 1210, 39);
R_Date("Luistari TU622  Hela-3961", 1199, 39);
R_Date("Luistari TU464  Hela-3968", 915, 34);
R_Date("Luistari TU465  Hela-3954", 1186, 34);
R_Date("Luistari TU471  Hela-3959", 873, 34);
R_Date("Luistari TU472  Hela-3963", 780, 39);
R_Date("Luistari TU473  Hela-3964", 1075, 36);
R_Date("Luistari TU478  Hela-3969", 1015, 36);
R_Date("Luistari TU480  Hela-3967", 839, 28);
R_Date("Luistari TU481  Hela-3973", 804, 34);
Span("Span of Luistari");
Interval("Duration");
};
Boundary("End of Luistari");
 };
```

Supplementary Figures S1a-S1g. Phase boundary outputs for each site containing four or more ^{14}C dates.

Phase boundaries were determined with Oxcal version 4.3 and IntCal 13 as the calibration curve for sites Levänluhta, Luistari, Hollola, Hiitola, Tuukkala, Pälkäne and Porvoo. Exact values are presented in Supplementary Table S2.

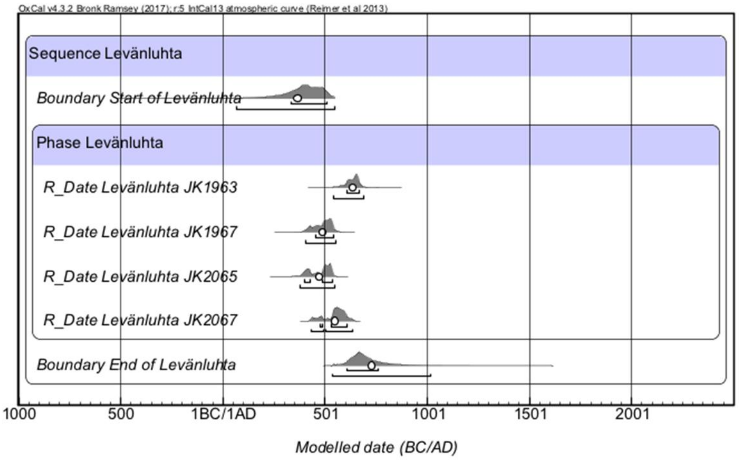

Supplementary Figure S1a. Phase's start and end boundaries determined for Levänluhta based on four ^{14}C datings. The mean value is presented with the circle. Under each individual probability distribution 68.2% and 95.4% rages are shown.

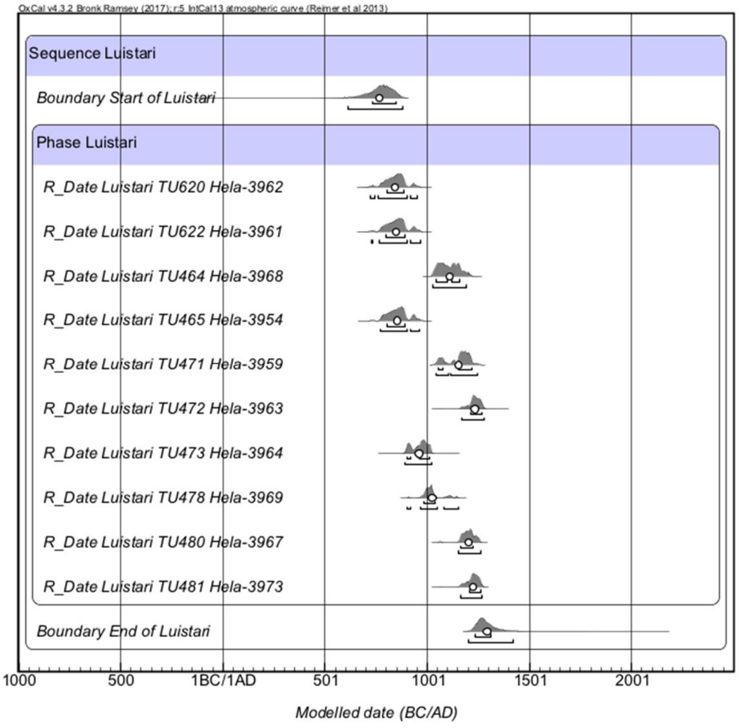

Supplementary Figure S1b. Phase's start and end boundaries determined for Luistari based on ten ^{14}C datings. The mean value is presented with the circle. Under each individual probability distribution 68.2% and 95.4% rages are shown.

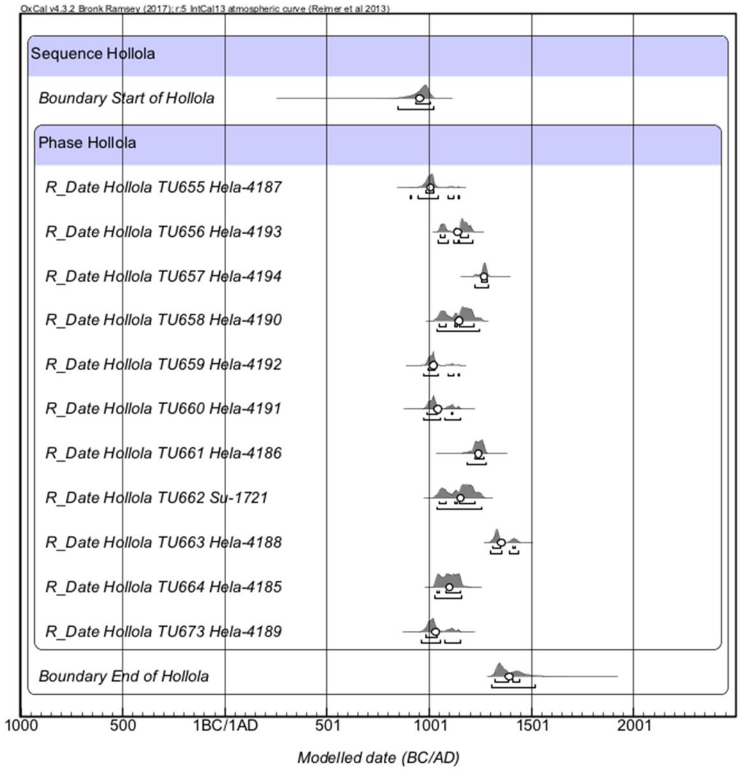

Supplementary Figure S1c. Phase's start and end boundaries determined for Hollola based on eleven ^{14}C datings. The mean value is presented with the circle. Under each individual probability distribution 68.2% and 95.4% rages are shown.

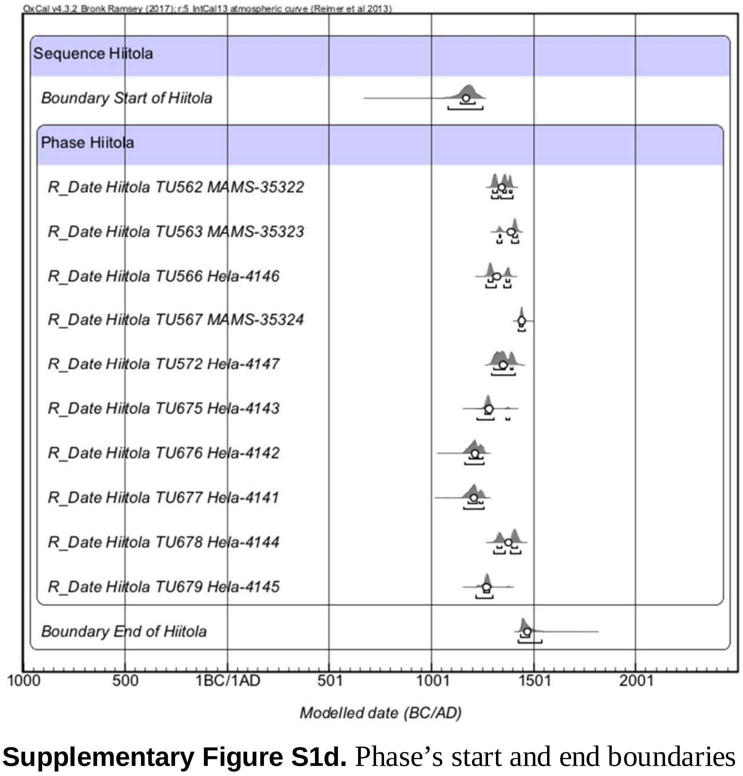

Supplementary Figure S1d. Phase's start and end boundaries determined for Hiitola based on ten ^{14}C datings. The mean value is presented with the circle. Under each individual probability distribution 68.2% and 95.4% rages are shown.

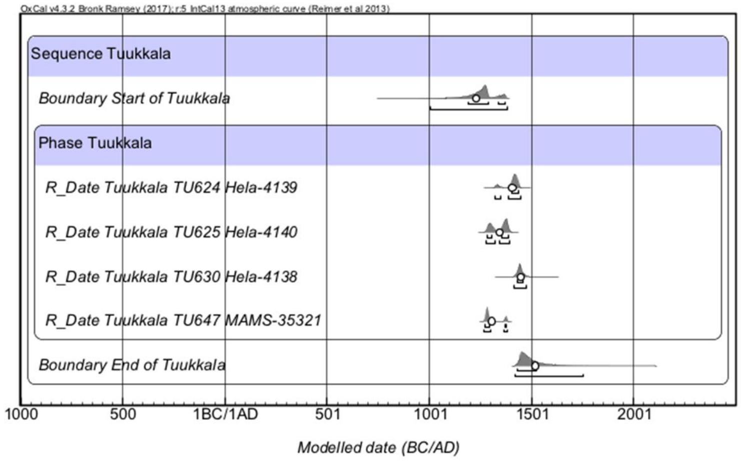

Supplementary Figure S1e. Phase’s start and end boundaries determined for Tuukkala based on four ^{14}C datings. The mean value is presented with the circle. Under each individual probability distribution 68.2% and 95.4% rages are shown.

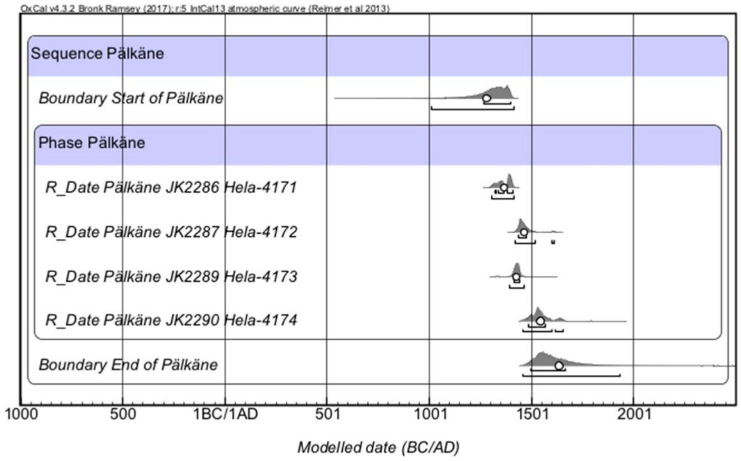

Supplementary Figure S1f. Phase’s start and end boundaries determined for Pälkäne based on four ^{14}C datings. The mean value is presented with the circle. Under each individual probability distribution 68.2% and 95.4% rages are shown.

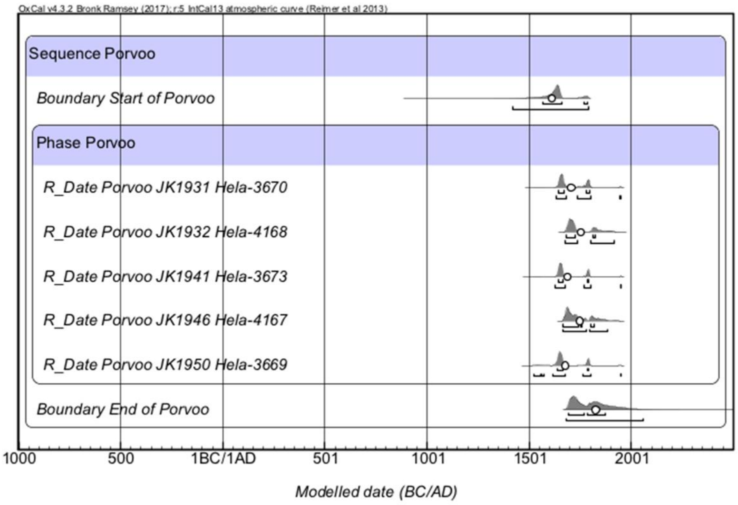

Supplementary Figure S1g. Phase's start and end boundaries determined for Porvoo based on five ^{14}C datings. The mean value is presented with the circle. Under each individual probability distribution 68.2% and 95.4% rages are shown.

Supplementary Figures S2a-S2e. Evaluation of the possible sampling bias in the observed haplogroup frequencies.

To evaluate whether sampling error (i.e., low sample sizes) alone could explain the observed differences in haplogroup frequencies in Iron-Age and medieval sites, simulation studies were performed by using the haplogroup frequencies observed in contemporary Finns as a reference (frequencies based on Neuvonen *et al.* 2015 [30]). Reference population was sampled N times according to the sample size of the ancient population of interest with 10000 permutations. Obtained distributions were compared with the observed haplogroup frequencies. For those haplogroups for which the observed frequency was less than 2.5 % or more than 97.5 % compared to the simulated frequency distribution, the observed frequency couldn't be explained by the sampling error alone (these haplogroups marked with red colour).

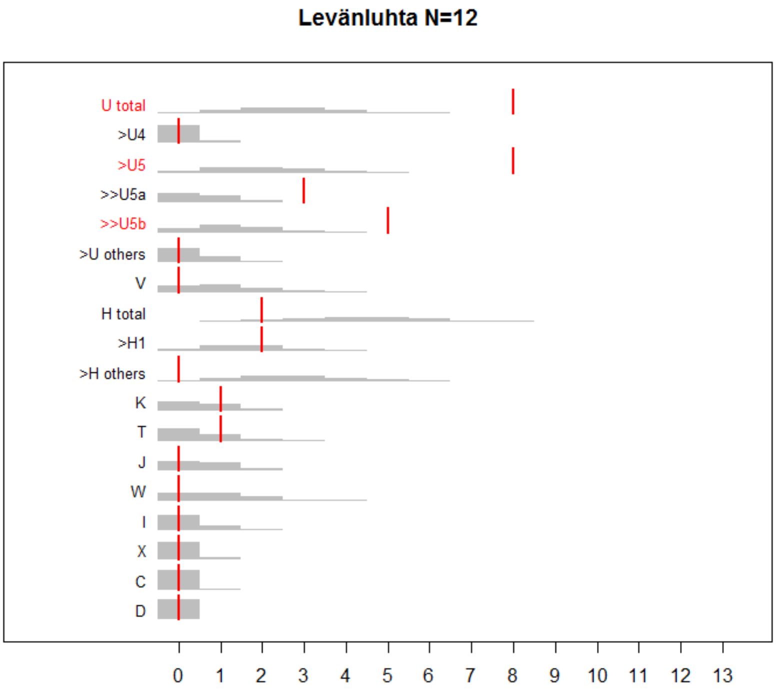

Supplementary Figure S2a. For Levänluhta frequencies for subhaplogroups U5a and U5b cannot be explained only by the sampling error.

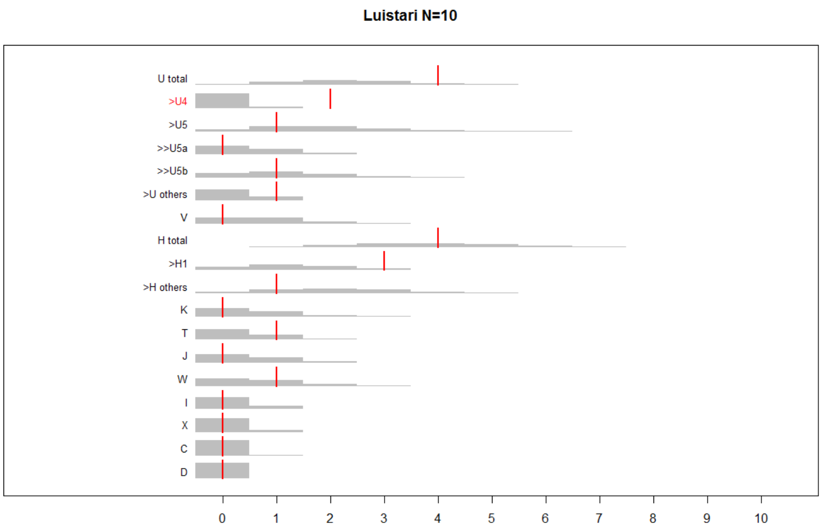

Supplementary Figure S2b. For Luistari frequency for subhaplogroup U4 cannot be explained only by the sampling error.

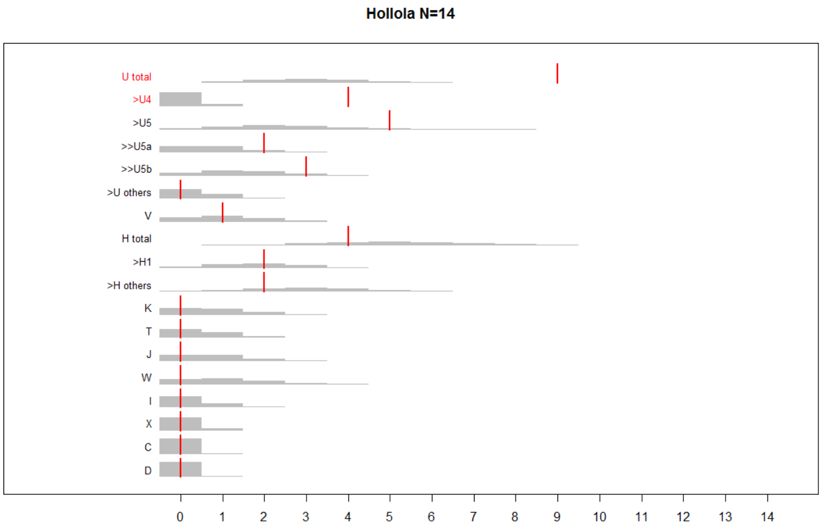

Supplementary Figure S2c. For Hollola frequency for subhaplogroup U4 cannot be explained only by the sampling error.

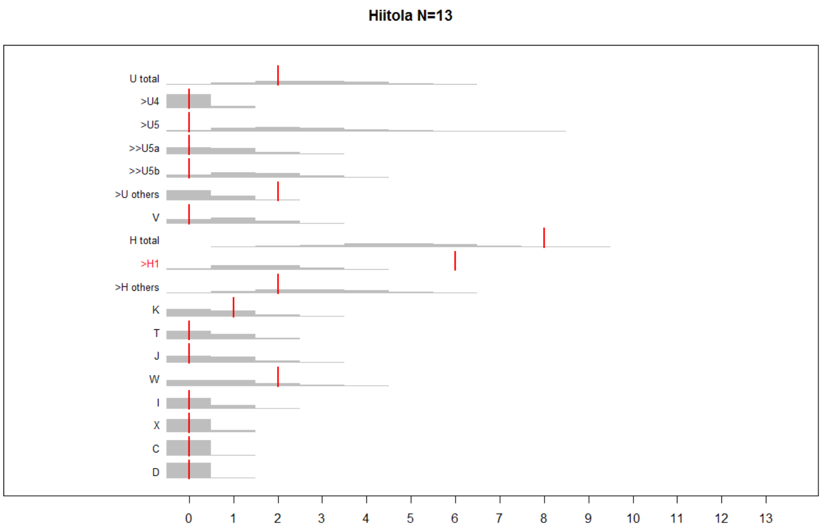

Supplementary Figure S2d. For Hiitola frequency for subhaplogroup H1 cannot be explained only by the sampling error.

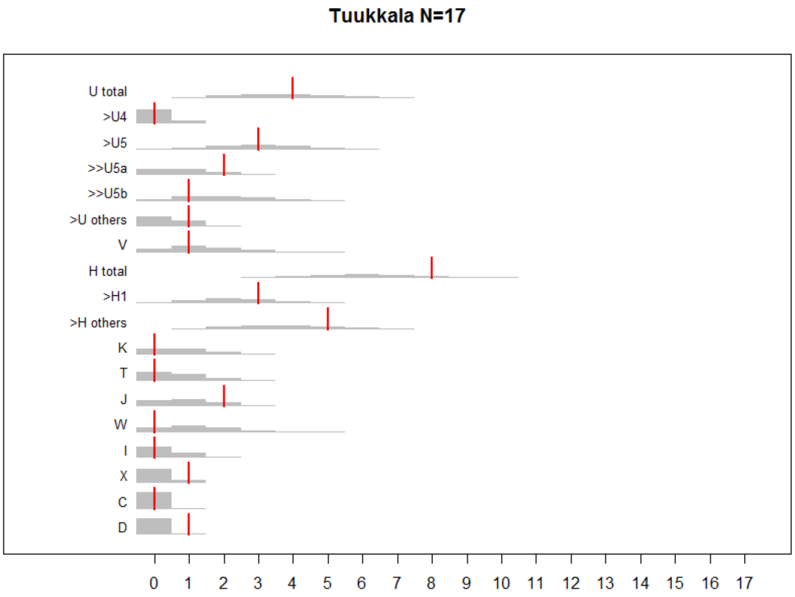

Supplementary Figure S2e. For Tuukkala observed haplogroup frequencies could be explained by the sampling error.

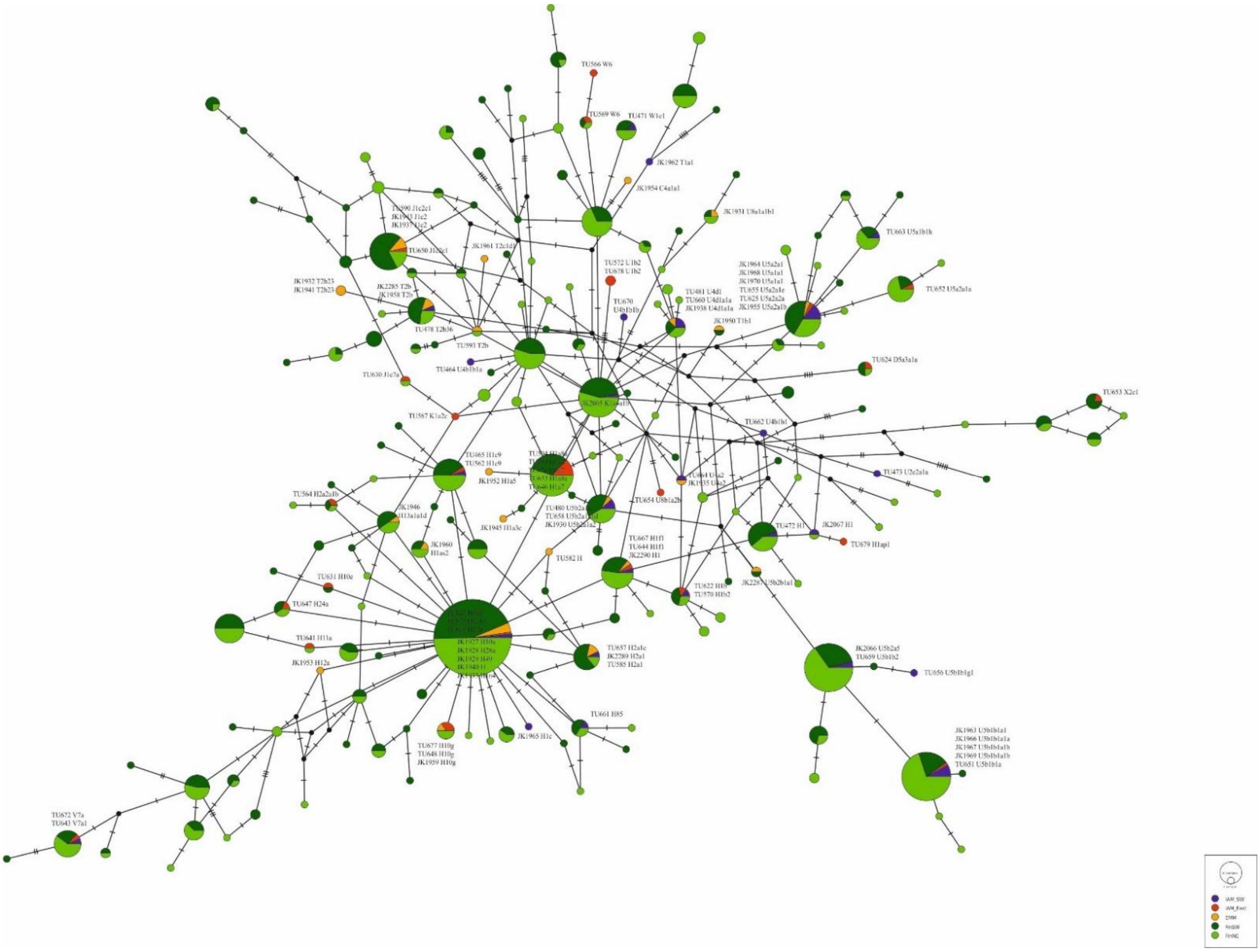

Supplementary Figure S3. Median-joining Network for ancient and contemporary Finns. Haplotype based median-joining network for ancient and contemporary Finns constructed based on HVR1+HVR2 data. Contemporary Finns obtained from Palo *et al.* 2009 [29] and Neuvonen *et al*. 2015 [30]. Iron Age and Medieval south-west = blue, Iron Age and Medieval East = red, Early modern and modern = yellow, contemporary south-west = dark green and contemporary north-east = light green.

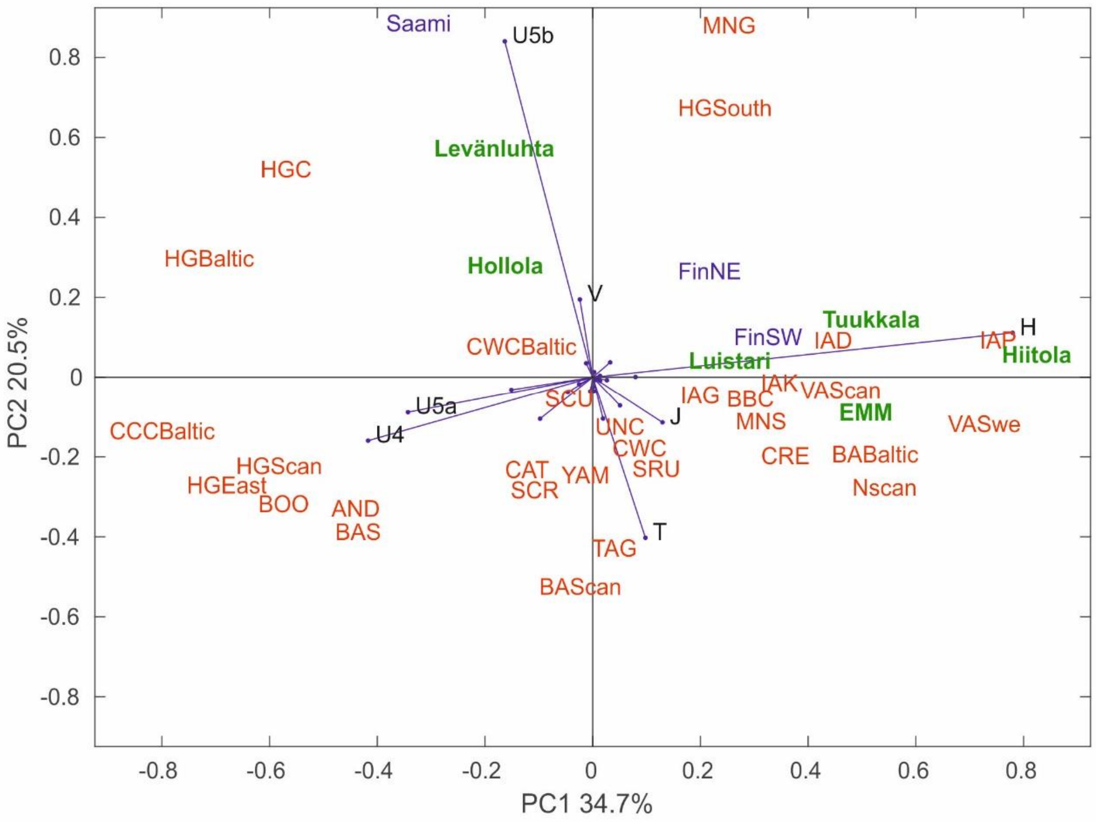

Supplementary figure S4. PCA biplot based on mitochondrial haplogroup frequencies with main loading vectors. Ancient populations presented in this study (green): Levänluhta, Luistari, Hollola, Hiitola, Tuukkala and Early modern and modern Finns (EMM). Contemporary populations (blue): north-east Finland (FinNE), south-west Finland (FinSW), Saami (SAA). Ancient populations (red): Andronovo culture (AND), Baltic Bronze-Age (BAB), Siberian Bronze-Age (BASi), Scandinavian Bronze-Age (BASc), Bell Beaker culture (BBC), Siberian Early Metal Period (BOO), Catacomb culture (CAT), Comb ceramic culture Baltic (CCCB), Crete Minoans (CRE), Corded Ware culture (CWC), Baltic Corded Ware Culture (CWCB), Baltic hunter-gatherers (HGB), central European hunter-gatherers (HGC), eastern hunter-gatherers (HGE), Scandinavian hunter-gatherers (HGSc), south European hunter-gatherers (HGSo), Denmark Iron-Age (IAD), Germany Iron-Age (IAG), Kazakhstan Iron-Age (IAK), Poland Iron-Age (IAP), Germany Middle-Neolithic (MNG), southern Europe Middle-Neolithic (MNS), Scandinavian Neolithic (NSc), Scythians from Russia (SCR), Scythians from Moldova and Ukraine (SCU), Srubnaya culture (SRU), Tagar culture (TAG), Unetice culture (UNC), Scandinavian Viking-Age (VASc), Sweden Viking-Age (VASw) ,Yamnaya culture (YAM).

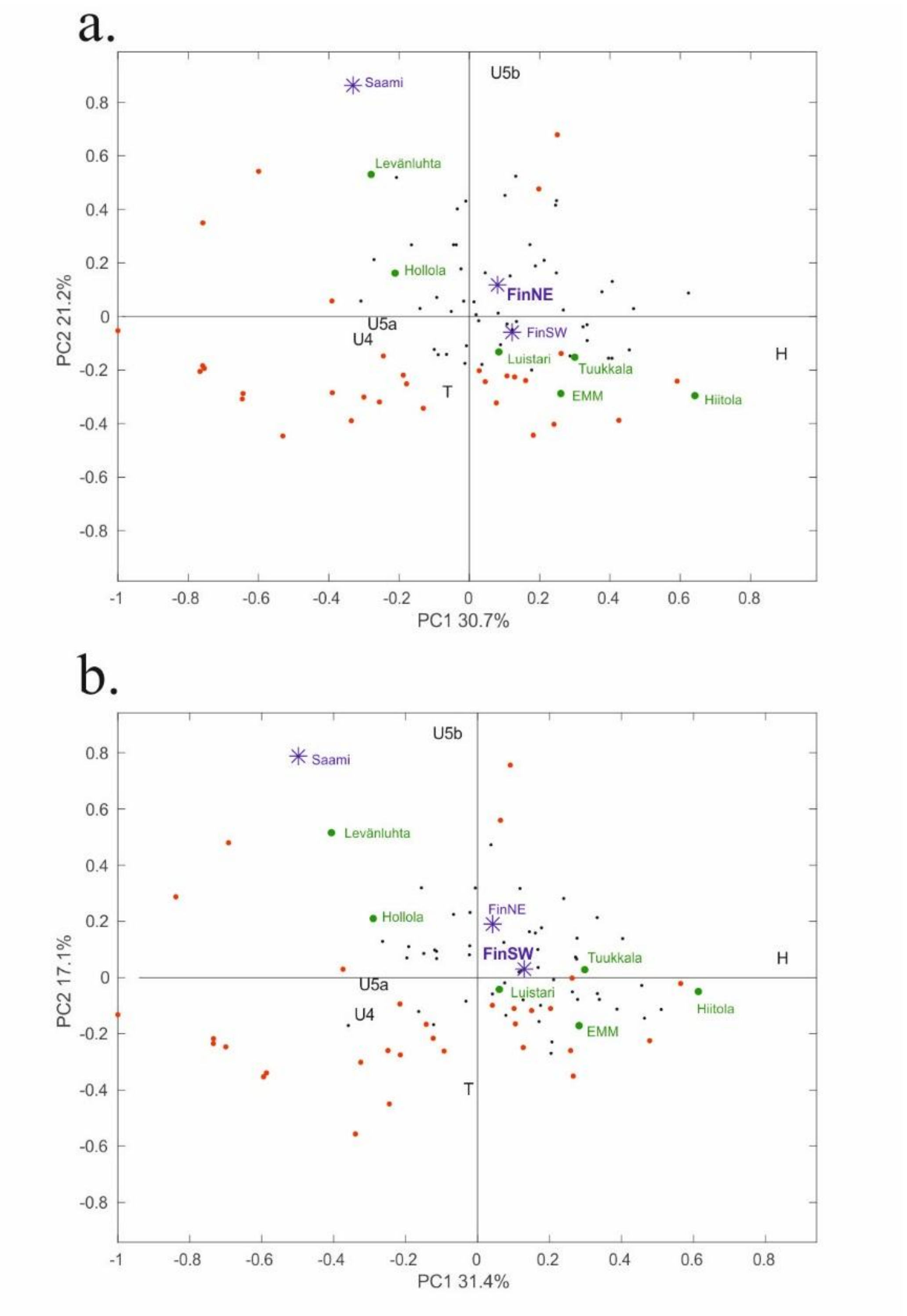

Supplementary Figure S5. The impact of small sample sizes on PCA. PCA was run with the observed ancient and modern data (as in Fig. 4 in the main article) as well as with fifty random samples (N=15 in each) drawn from contemporary a) northeastern (N = 443), b) southeastern (N = 389) Finns. Black dots denote the locations of these random samples. The scattering of these dots reveal substantial effect of limited sample sizes on the PCA. Blue asterisks: contemporary northeastern Finns (FinNE), southwestern Finns (FinSW) and Saami, green dots: ancient populations presented in this study (Levänluhta, Luistari, Hollola, Hiitola, Tuukkala and Early modern and modern Finns (EMM)); red dots: other ancient populations.

References

1. T. Formisto, An Osteological Analysis of Human and Animal Bones from Levänluhta (Vammalan Kirjapaino, 1993)

2. A. Wessman, Levänluhta. A place of punishment, sacrifice or just a common cemetery. Fennoscandia Archaeologica 26, 81–105 (2009)

3. A. Wessman, T. Alenius, E. Holmqvist, K. Mannermaa, W. Perttola, T. Sundell, S. Vanhanen, Hidden and Remote: New perspectives on the people in the Levänluhta water burial, Western Finland (c. AD 300-800). European Journal of Archaeology 21, 431–454 (2018)

4. P-L. Lehtosalo-Hilander, Luistari in Eura. From pagan burial-ground to Christian cemetery. Abhandlungen der Geistes-und Sozialwissenschaftlichen Klasse-Akademie der Wissenschaften und der Literatur 3, 389–403 (1997)

5. K. Uotila, Eura, Kauttua. Luistarintien kaivaukset 29.7.–7.10.2013. Muuritutkimus ky. (Excavation report in Finnish) https://www.kyppi.fi/palveluikkuna/raportti/read/asp/hae_liite.aspx?id=122587&ttyyppi=pdf&kansio_id=50 (2014)

6. P-L. Lehtosalo-Hilander, Luistari I (The Graves), II (The Artefacts) & III (An Inhumanition Burial-ground Reflecting the Finnish Viking Age Society) (Suomen Muinaismuistoyhdistyksen Aikakausikirja 82, Vammalan Kirjapaino, Vammala, 1982)

7. P-L. Lehtosalo-Hilander, Luistari: A History of Weapons and Ornaments (Suomen Muinaismuistoyhdistyksen Aikakausikirja 107, Vammalan Kirjapaino, Vammala, 2000)

8. A-L. Hirviluoto, in Hollolan Kirkko- Asutuksen, Kirkon ja Seurakunnan Historiaa (Hollolan seurakunta, Hollola, 1985), pp.8–36

9. J-P. Taavitsainen, Ancient Hillforts of Finland: Problems of Analysis, Chronology, and Interpretation with Special Reference to the Hillfort of Kuhmoinen (Suomen Muinaismuistoyhdistyksen Aikakausikirja 94, Ekenäs Tryckeri ab, Ekenäs, 1990)

10. P. Sarvas, Ristiretkiajan Ajoituskysymyksiä (Suomen Museo, 1971)

11. K. Salo, PhD Thesis, University of Helsinki (2016) Thesis available online https://helda.helsinki.fi/handle/10138/163042

12. J. Ailio, Karjalaiset Soikeat Kupurasoljet: Katkelmia Karjalan Koristetyylin Kehityshistoriasta (Suomen Muinaismuistoyhdistyksen Aikakausikirja 32, Suomen Muinaismuistoyhdistys, Helsinki, 1922)

13. E. Tõnisson, in Eesti Esiajalugu, (Eesti Raamat, Tallinn, 1982), pp. 355

14. E. Kivikoski, Die Eisenzeit Finnlands: Bildwerk und Text (Suomen Muinaismuistoyhdistys, Helsinki, 1973)

15. S. Bel'skiy, V. Laakso, in Rome, Constantinople and Newly-Converted Europe: Archaeological and Historical Evidence, M. Salamon, M. Wołoszyn, A. Musin, P. Špehar, M. Hardt, M.P. Kruk, A. Sulikowska-Gąska, Eds. (U źródeł Eu ropy Środkowo-ws chodniej/Frühzeit Ostmitteleuropas, 2012), pp. 767–775

16. P. Purhonen, Maiseman Muisti: Valtakunnallisesti Merkittävät Muinaisjäännökset (Museovirasto, 2001)

17. E. Mikkola, The Mikkeli Tuukkala cemetery - the 2009 excavation and new interpretations. Fennoscandia Archaeologica 26, 177–185

18. P-L. Lehtosalo-Hilander, in Savon Historia I: Esihistorian Vuosituhannet Savon Alueella ja Savon Keskiaika, P-L. Lehtosalo-Hilander, K. Pirinen, Eds. (Kustannuskiila, Kuopio, 1988), pp. 217–218

19. E. Mikkola, K. Vuoristo, Pälkäneen Rauniokirkko, Myöhäiskeskiaikaisen kivikirkon kaivaus 4.6–4.7.2003 ja 8.9–19.9.2003, Museovirasto (Excavation report in Finnish) https://www.kyppi.fi/palveluikkuna/raportti/read/asp/hae_liite.aspx?id=118133&ttyyppi=pdf&kansio_id=635 (2004)

20. K. Vuoristo, Pälkäne Rauniokirkko, Myöhäiskeskiaikaisen kivikirkon asehuoneen kaivaus 31.5–18.6.2010 ja 21–22.7.2010, Museovirasto (Excavation report in Finnish) https://www.kyppi.fi/palveluikkuna/mjhanke/read/asp/hae_liite.aspx?id=113663&ttyyppi=pdf&kansio_id=635 (2011)

21. M. Hiekkanen, Suomen Keskiajan Kivikirkot (Suomalaisen Kirjallisuuden Seura, Helsinki, 2014)

22. J. Lagerstedt, Porvoon tuomiokirkon kirkkomaa, arkeologinen kaivaus, Museovirasto/RHO (Excavation report in Finnish) https://www.kyppi.fi/palveluikkuna/raportti/read/asp/hae_liite.aspx?id=118055&ttyyppi=pdf&kansio_id=638 (2008)

23. C.J. Gardberg, in Porvoon Kaupungin Historia I: Porvoon Seudun Esihistoria, Keskiaika ja 1500-luku, T. Edgren, C.J. Gardberg, Eds. (WSOY, 1996), pp. 200–205

24. I. Mäntylä, Porvoon Kaupungin Historia II: 1602–1809 (WSOY, 1994)

25. M. Hiekkanen, in Rengon Historia, M. Hiekkanen, J. Härme, Eds. (Gummerus, Jyväskylä, 1993), pp. 52–252

26. J. Härme, in Rengon Historia, M. Hiekkanen, J. Härme, Eds. (Gummerus, Jyväskylä, 1993), pp. 479–489

27. S. Pihlman, in Finskt Museum, M. Schauman-Lönnqvist, Eds. (Finska Fornminnesföreningen, Vammala, 1994)

28. K. Vuoristo, Hamina rykmentinkenttä, historiallisen ajan arkeologinen kaupunkikaivaus 5.5–12.8.2011, Museovirasto (Excavation report in Finnish) https://www.kyppi.fi/palveluikkuna/mjhanke/read/asp/hae_liite.aspx?id=114380&ttyyppi=pdf&kansio_id=75 (2012)

29. J.U. Palo *et al.* Genetic markers and population history: Finland revisited. *Eur. J. Hum. Genet.* 17, 1336–1346 (2009).

30. A. Neuvonen *et al.* Vestiges of an Ancient Border in the Contemporary Genetic Diversity of North-Eastern Europe. *PLoS ONE* 10, 1-19 (2015).
